# Superoxide Dismutases, SOD1 and SOD2, Play a Distinct Role in the Fat Body during Pupation in Silkworm *Bombyx mori*

**DOI:** 10.1371/journal.pone.0116007

**Published:** 2015-02-25

**Authors:** Yosui Nojima, Katsuhiko Ito, Hiromasa Ono, Takeru Nakazato, Hidemasa Bono, Takeshi Yokoyama, Ryoichi Sato, Yoshitaka Suetsugu, Yuki Nakamura, Kimiko Yamamoto, Jun-ichi Satoh, Hiroko Tabunoki, Hajime Fugo

**Affiliations:** 1 Department of Biological Production, Faculty of Agriculture, Tokyo University of Agriculture and Technology, Saiwai-cho 3-5-8, Fuchu, Tokyo 183-8509, Japan; 2 Department of Bioinformatics and Molecular Neuropathology, Meiji Pharmaceutical University, Noshio, 2-522-1, Kiyose, Tokyo 204-8588, Japan; 3 Database Center for Life Science (DBCLS), Research Organization of Information and Systems (ROIS), Yata 1111, Mishima, Shizuoka 411-8540, Japan; 4 Bio-Applications and Systems Engineering, Tokyo University of Agriculture and Technology, Koganei, Tokyo 184-8588, Japan; 5 Insect Genome Research Unit, National Institute of Agrobiological Sciences, Tsukuba 305-8634, Japan; Institute of Plant Physiology and Ecology, CHINA

## Abstract

One way that aerobic biological systems counteract the generation of reactive oxygen species (ROS) is with superoxide dismutase proteins SOD1 and SOD2 that metabolize superoxide radicals to molecular oxygen and hydrogen peroxide or scavenge oxygen radicals produced by the extensive oxidation-reduction and electron-transport reactions that occur in mitochondria. We characterized SOD1 and SOD2 of *Bombyx mori* isolated from the fat body of larvae. Immunological analysis demonstrated the presence of BmSOD1 and BmSOD2 in the silk gland, midgut, fat body, Malpighian tubules, testis and ovary from larvae to adults. We found that BmSOD2 had a unique expression pattern in the fat body through the fifth instar larval developmental stage. The anti-oxidative functions of BmSOD1 and BmSOD2 were assessed by exposing larvae to insecticide rotenone or vasodilator isosorbide dinitrate, which is an ROS generator in BmN4 cells; however, exposure to these compounds had no effect on the expression levels of either BmSOD protein. Next, we investigated the physiological role of BmSOD1 and BmSOD2 under environmental oxidative stress, applied through whole-body UV irradiation and assayed using quantitative RT-PCR, immunoblotting and microarray analysis. The mRNA expression level of both BmSOD1 and BmSOD2 was markedly increased but protein expression level was increased only slightly. To examine the differences in mRNA and protein level due to UV irradiation intensity, we performed microarray analysis. Gene set enrichment analysis revealed that genes in the insulin signaling pathway and PPAR signaling pathway were significantly up-regulated after 6 and 12 hours of UV irradiation. Taken together, the activities of BmSOD1 and BmSOD2 may be related to the response to UV irradiation stress in *B*. *mori*. These results suggest that BmSOD1 and BmSOD2 modulate environmental oxidative stress in the cell and have a specific role in fat body of *B*. *mori* during pupation.

## Introduction

Reactive oxygen species (ROS) are constantly generated in all aerobic biological systems as the natural products of oxidative metabolism and are also produced by the exposure of tissues and cells to environmental stress, extreme temperatures and chemical agents. In living organisms, the narrowly defined ROS are known as superoxide anions (O_2_^−^), hydroxyl radical (HO), hydrogen peroxide (H_2_O_2_) and singlet oxygen (^1^O_2_) and they are generated by exposure to ultraviolet (UV) irradiation or chemical agents such as mitochondria complex I inhibitors [1–3]. The broadly defined ROS include nitric oxide (NO), lipid peroxide and ozone (O_3_). ROS are toxic to living organisms due to their high reactivity, which causes oxidative damage to proteins, lipids and nucleic acids, and ROS are related to aging and lifespan [4,5]. However, studies using *Drosophila* have shown that ROS not only act as destructive molecules but are also involved in cell signaling networks [6]. Therefore, the balance between the generation and elimination of ROS in the cell is important. Superoxide dismutase (SOD) proteins play a role in removing ROS by catalyzing disproportionation to O_2_ and hydrogen peroxide, after which hydrogen peroxide is converted into water by catalase or glutathione peroxidase [7].

Three kinds of SOD proteins have been reported to date. SOD1 is a major cytoplasmic antioxidant enzyme that metabolizes superoxide radicals to molecular oxygen and hydrogen peroxide, thus providing a defense against oxygen toxicity. Soluble cytoplasmic SOD1 is a copper- and zinc-containing enzyme (Online Mendelian Inheritance in Man; OMIM, 147450). SOD2 is a mitochondrial matrix enzyme that scavenges oxygen radicals produced by the extensive oxidation-reduction and electron transport reactions that occur in mitochondria (OMIM, 147460). These SOD proteins belong to the family of metalloenzymes and are widely distributed in prokaryotes and eukaryotes, being classified as copper/zinc SOD (Cu/Zn SOD; SOD1) and manganese SOD (Mn SOD; SOD2) [8]. In addition, an extracellular form of SOD protein has been identified. EC-SOD (SOD3) is found in the plasma, lymph and synovial fluid, as well as in tissues (OMIM, 185490) of vertebrates and invertebrates.

The silkworm *Bombyx mori*, a lepidopteran insect, has been utilized as a model insect for agricultural research for several reasons: the majority of agricultural pests are lepidopteran insects, its genome is well characterized, various genetic mutants are available, and it is compatible with transgenic, knock-out and microarray technologies [9–13]. The complete silkworm genome has 16,823 gene loci, including 5,748 human orthologs [14]. In *B*. *mori*, three SODs were found: SOD1, SOD2 and SOD3. Of these, SOD3 was found to control egg diapause status, and it was named the time interval measuring enzyme-esterase A4 (TIME-EA4) [15]. While cDNA sequences of *B*. *mori* SOD1 and SOD2 have been reported, their products and biological functions are unclear [16,17].

We characterized the functions of *B*. *mori* SOD1 and SOD2 proteins using the hybrid strain of the domestic silkworm Kinshu x Showa, which has a larger larval body size than other domestic silkworm strains and can easily reared in any season. Thus, the Kinshu x Showa is easy to work with and is suitable for use in biochemical and physiological experiments such as these.

## Materials and Methods

### Insects

The *B*. *mori* hybrid strain Kinshu x Showa supplied by Ueda-Sha Co. Ltd. (Nagano, Japan) was used in all experiments. Silkworm larvae were reared on the artificial diet silk-mate 2S (Nosan, Tsukuba, Japan). All larvae were kept at 25°C on a 12-hour light/12-hour dark cycle.

### Cell culture

A silkworm cell line, BmN4 (Sysmex Co. Ltd., Saitama, Japan) derived from ovary, was maintained at 25°C in TC-100 medium (Appli Chem Co., Ltd., Darmstadt, Germany) supplemented with 10% fetal bovine serum and antibiotic-antimycotic (Invitrogen, Carlsbad, CA).

### UV irradiation

Day 3 fifth instar larvae were treated with ultraviolet (UV) rays using UVL-56 (1350 μW/cm^2^, UVP) for 1, 2, 6 and 12 hours (4.86, 9.72, 29.2 and 58.32 J/cm^2^). We prepared a cardboard box with two stands at either side to position two UVL-56 lamps at the top of the box (S1 Fig.). Irradiation distance was adjusted to 7.5cm from the face of the UVL-56 lamps to inside of the plastic box. Silkworm larvae were placed on the inside of the box for UV irradiation, after which they were fed artificial diet for 16 hours until dissection of the fat body.

### Microarray analysis

Total RNA was isolated from the fat body of day 3 fifth instar non-irradiated and UV-irradiated (29.2 and 58.32 J/cm^2^) larvae using RNeasy Mini Kits (Qiagen, Valencia, CA) and quantified on an Agilent 2100 Bioanalyzer (Agilent Technologies, Palo Alto, CA). Microarrays of Cy3-labeled cRNA were prepared for non-irradiated and UV-irradiated larvae (n = 5 each). Briefly, 300 ng of total RNA was processed to Cy3-labeled cRNA using an Agilent Quick Amp Labeling Kit (Agilent Technologies) according to manufacturer instructions followed by purification with an RNeasy Mini Kit (Qiagen) and quantification on an Agilent 2100 Bioanalyzer (Agilent Technologies) and a NanoDrop 1000 spectrophotometer (Thermo Scientific, Waltham, MA). Hybridization was performed using a Gene Expression Hybridization Kit (Agilent Technologies) for which 1.65 μg of Cy3-labeled cRNA was mixed, fragmented and hybridized on a silkworm 4 × 44 K custom oligo-microarray slide containing 43,864 spots of 60-mer oligonucleotides constructed from 17,615 EST silkworm sequences (Agilent Technologies) and incubated at 65°C for 17 hours with shaking at 10 rpm. UV-irradiated and non-irradiated (control) samples were applied to a single array slide. The arrays were washed in Agilent Gene Expression Wash Buffer 1 (Agilent Technologies) for 1 min at room temperature and Agilent Gene Expression Wash Buffer 2 (Agilent Technologies) for 1 min at 37°C. The intensity of hybridized probes was detected with an Agilent G2565BA Microarray Scanner (Agilent Technologies) set to 5-μm scan resolution, and signals were extracted with G2565AA Feature Extraction Software v.9.5 (Agilent Technologies). Raw data collected in text files were normalized ‘per spot’ and ‘per chip’ using the GENESPRING GX v.10.3 program (Agilent Technologies). The microarray data discussed in this publication have been deposited in NCBI’s Gene Expression Omnibus (GEO) database under GEO accession numbers GSM1346427, GSM1346428, GSM1346429 and GSM1346430 (series GSE55816).

### Microarray Data analysis

TIBCO Spotfire (TIBCO Software, Inc., Somerville, MA, USA) was used for hierarchical clustering and identification of gene sets differentially expressed in the UV irradiation treatment. Differential expression analysis for gene sets after UV irradiation was also performed with TIBCO Spotfire. Differences in gene expression greater than 2-fold and with P-values less than 0.05 were considered to be significant. The silkworm-retrieved gene sets that were up- and down-regulated by UV irradiation were converted to corresponding human homologous genes by a systematic BLAST search (tblastx), as described previously [18]. Gene set enrichment analysis (GSEA) was then performed for up- and down-regulated gene sets for the UV-irradiated mRNA expression profiles. A database for annotation, visualization and integrated discovery (DAVID) functional annotation tools [19, 20] was used to identity UV-irradiation-specific gene sets in the KEGG pathway and Gene Ontology (GO) biological process category.

### Preparation of recombinant BmSOD1 and BmSOD2

Primers for PCR were designed from BmSOD1 and BmSOD2 cDNA sequences obtained from GenBank and RefSeq (BmSOD1, Accession no. AB179561; BmSOD2, AB190802). Total RNA was extracted from the fat body of day 3 fifth instar larvae using an RNeasy mini kit (Qiagen). DNase-treated total RNA was processed for cDNA synthesis using oligo(dT)12–18 primers and SuperScript II reverse transcriptase (Invitrogen). The ORFs of BmSOD1 and BmSOD2 were amplified by PCR using PfuTurbo DNA polymerase (Agilent Technologies) with the following primers: BmSOD1, 5′-CCCGCCAAAGCAGTTTGCGTACTTC-3′ and 5′-TTAAATCTTGGCCAAGCCAATGACT-3′; BmSOD2, 5′-TTAATGTCACAAAGGATTGGATCA-3′ and 5′-TCACTTGAGCGCTTTTTCATATCT-3′. Products were cloned into prokaryotic expression vector pTrcHis-TOPO using a TOPO TA cloning kit (Invitrogen) and were expressed in *Escherichia coli* XL-1 blue strain as fusion proteins with N-terminal Xpress tags. The nucleotide sequence was confirmed by DNA sequencing. Recombinant BmSOD1 and BmSOD2 expressed in *E*. *coli* were purified with HIS-Select spin columns (Sigma, St. Louis, MO) according to supplier instructions.

### Immunoblotting

First, we examined the specificity of antibodies against BmSOD1 and BmSOD2 using the following samples: 10 μg of HeLa cell lysate, 10 μg of BmN4 cell lysate, 10 μg of larvae testis lysate, 0.5 μl of recombinant BmSOD1 with Xpress-tag, and 0.1 μl of recombinant BmSOD2 with Xpress-tag as a positive control. Also, the tissue distribution of BmSOD1 and BmSOD2 was determined for the silk gland, midgut, fat body, Malpighian tubules, testis and ovary from day 3 fifth instar larvae or the fat body from day 0 to 6 fifth instar larvae. The distribution of BmSOD1 and BmSOD2 in the whole body from stages including first to fifth instar larvae, pupae and adults was also determined. All tissues were homogenized in RIPA lysis buffer composed of 50 mM Tris-HCl, pH 7.5, 150 mM NaCl, 1% Nonidet P40, 0.5% sodium deoxycholate, 0.1% SDS (Sigma-Aldrich, St. Louis, MO, USA) and protease inhibitor cocktail (Roche Diagnostics, Mannheim, Germany), followed by centrifugation at 10,000 × g for 15 min. Protein concentration was determined by a BCA protein assay kit (Thermo Scientific Co., Ltd., Rockford, IL). To identify the presence of BmSOD1 and BmSOD2 in different tissues, protein samples (10 μg) were separated on SDS-PAGE, transferred to nitrocellulose membranes using the method of Towbin et al. [21], and immunoblotted using rabbit anti-SOD1 antibody 1:2000, for BmSOD1 (ab13498; Abcam Cambridge, UK), rabbit anti-SOD2 antibody 1:2000, for BmSOD2 (ab13534; Abcam) and goat anti-rabbit IgG-conjugated horseradish peroxidase (HRP) 1:2000 (sc-2004; Santa Cruz Biotechnology, Santa Cruz, CA) Membranes were developed using a chemiluminescent substrate (Pierce, Rockford, IL).

After antibodies were stripped by incubating the membranes at 50°C for 30 min in stripping buffer composed of 62.5 mM Tris–HCl, pH 6.8, 2% SDS and 100 mM 2-mercaptoethanol, membranes were processed for relabeling with different antibodies. Protein levels were measured by ImageJ ver. 1.37 c (http://imagej.nih.gov/ij/index.html).

### Quantitative RT-PCR

In order to quantify RNA expression levels, total RNA was extracted from pooled fat body tissue dissected from day 3 fifth instar larvae (n = 3–5 each) using an RNeasy Mini Kit (Qiagen). One-step RT-PCR was performed in 20 μl reaction volumes with 1 μg of RNA template and custom-made primers and probes (Table 1) with a TaqMan RNA-to-CT 1-Step Kit (Applied Biosystems, Foster City, CA), in accordance to manufacturer instructions. Quantitative RT-PCR (qRT-PCR) was performed on a 7500 Fast Real-Time PCR System (Applied Biosystems) following the Delta-Delta Ct method. Actin was utilized as an endogenous reference against which RNA expression levels were standardized, and all data were calibrated against universal reference data. Relative quantification (RQ) values represent the relative expression level against a reference sample. All sample sets were assayed in triplicate as technical replications.

**Table 1:** Primers and probes used for quantitative RT-PCR.

Gene	Probe	Forward primer	Reverse primer
BmSOD1	5′-TACGCCATGTCGGCGACCTCG-3′	5′-ATCATGGTGGTCCCAGTTCTG-3′	5′-CAGAGTCTTCAATTGCCTCAATGT-3′
BmSOD2	5′-CCACTCGATCTTTTGGCACAACCTGT-3′	5′-TCAATGGTGGTGGTCACATCA-3′	5′-AGGCTTGCCACCATTTGG-3′
BmActin	5′- AAGGTTACGCTCTGCCCCACGCC-3′	5′-CTCCCACACCGTACCCATCT-3′	5′-AAGTCGCGACCAGCCAAGT-3′

Probes and primer sets were custom designed with 5’ labeled 6 FAM and 3’ labeled TAMRA.

### Northern blot analysis

Total RNA derived from the fat body of day 3 fifth instar larvae was used. Total RNA (12 μg) was separated on a 1.5% agarose, 6% formaldehyde gel and stained with ethidium bromide. Then, the gel was transferred to a nylon membrane. DIG-labeled probes were synthesized using the PCR DIG probe synthesis kit (Roche Diagnostics, Mannheim, Germany) in accordance with supplier instructions using the following primers: BmSOD1, 5′-CACGAATTTGGTGACAACACAAATG-3′ and 5′-TTAAATCTTGGCCAAGCCAATGACT-3′; and BmSOD2, 5′-ATCAACTGTCGACAGCTTCTGT-3′ and 5′-TCACTTGAGCGCTTTTTCATA-3′. After pre-hybridization, membranes were hybridized with DIG-labeled probes at 50°C overnight. The specific reaction was visualized on Kodak XOMAT AR X-ray film using a DIG chemiluminescence detection kit (Roche Diagnostics). 18S ribosomal RNA (rRNA) was used as a control.

The size of the mRNA for both SODs was calculated using image analysis software CS Analyzer 3.0 (ATTO, Tokyo, Japan). A calibration curve was determined using the mobility of the DIG RNA ladder marker (Roche Diagnostics).

### BmN4 cells treated with ROT or ISDN and detection of BmSOD1 and BmSOD2

BmN4 cells (2 × 10^6^) were grown on 6-well Falcon plates (BD Biosciences, Franklin Lakes, NJ) and washed twice with PBS followed by treatment with TC-100 medium containing rotenone (ROT; 50 μM) dissolved in 0.1% DMSO or 100 μM of isosorbide dinitrate (ISDN; prepared immediately prior to use and kept in the dark) dissolved in 0.1% ethanol for 3 or 6 hours. Control experiments were performed with either 0.1% DMSO or 0.1% ethanol. Total protein extracts were prepared using RIPA buffer (Sigma-Aldrich) for immunoblotting.

### Statistical analysis

Student’s t-test was carried out using JMP 10.0 software (SAS Institute Japan Ltd., Tokyo, Japan), and P values of <0.05 were considered significant.

### Bioinformatics analysis

A search for SOD1 and SOD2 orthologs in the genomes of *Plutella xylostella* (http://dbm.dna.affrc.go.jp/px/) and *Manduca sexta* (http://agripestbase.org/manduca/?q=download) were conducted using BLAST methods. Global homology searches were conducted using Genetyx ver. 11 (Genetyx Co. Ltd., Tokyo, Japan). Phylogenic analysis was performed using Clustal W ver. 2.1 (http://clustalw.ddbj.nig.ac.jp/). A protein motif search was conducted using SMART (http://smart.embl-heidelberg.de/). Alignment of the deduced BmSOD1 and BmSOD2 amino acid sequences and SOD1 and SOD2 orthologs from other species was conducted using CLC Sequence viewer 6.8 (CLC Bio Japan Inc. Tokyo, Japan).

## Results

### cDNA cloning and sequence analysis of BmSOD1 and BmSOD2

Using RNA isolated from the fat body of day 3 fifth instar larvae, the deduced ORF of BmSOD1 was 465 nucleotides coding for a protein with 154 amino acids, a molecular weight of 15,841 Da and a putative isoelectric point (pI) of 5.78, while the deduced ORF of BmSOD2 was 651 nucleotides coding for a protein with 216 amino acids, a molecular weight of 24,226 Da, and a putative isoelectric point (pI) of 9.18. A protein motif search revealed that BmSOD1 contains a copper/zinc superoxide dismutase domain (SOD_Cu, pfam; PF00080) at position 5A-149G and BmSOD2 contains the following domains: iron/manganese superoxide dismutases alpha-hairpin domain (Sod_Fe_N, pfam; PF00081) at position 20R-101N and an iron/manganese superoxide dismutase c-terminal domain (Sod_Fe_C, pfam; PF02777) at 105F-211V. Thus, both SODs were successfully cloned from the fat body of *B*. *mori* larvae. We localized the BmSOD1 gene to four blocks between bp 356678 and 360180 on chromosome 23 and the BmSOD2 gene to six blocks between bp 916397 and 921150 on chromosome 3 by linkage mapping with 28 chromosomes using SNP markers [22].

BmSOD1 and BmSOD2 have greater than 60% amino acid sequence identity to vertebrate SOD1 and SOD2 proteins, including those from *Rattus norvegicus*, *Homo sapiens*, *Mus musculus*, *Danio rerio* and *Xenopus tropicalis*. BmSOD1 and BmSOD2 also show high homology to insect SOD1 and SOD2 proteins, including those from *Anopheles gambiae*, *Drosophila melanogaster*, *Plutella xylostella* and *Manduca sexta* (Tables 2 and 3). In particular, BmSOD1 and BmSOD2 showed high similarity to proteins from lepidopteran insects.

**Table 2:** Amino acid sequence identity among BmSOD1 and other SOD1 homologs.

Species	GeneID	Identity (%)
*Rattus norvegicus*	NP_058746.1	63
*Homo sapiens*	NP_000445.1	64
*Mus musculus*	NP_035564.1	64
*Danio rerio*	NP_571369.1	64
*Xenopus tropicalis*	NP_001016252.1	65
*Anopheles gambiae*	XP_311594.2	65
*Drosophila melanogaster*	NP_476735.1	68
*Plutella xylostella*	PXGS_V2_006641, 031456	79
*Manduca sexta*	Msex000492-RA	86

Gene ID represents the NCBI reference sequence ID, except for *P*. *xylostella* and *M*. *sexta* which are gene IDs from their respective databases.

**Table 3:** Amino acid sequence identity among BmSOD2 and other SOD2 homologs.

Species	GeneID	Identity (%)
*Rattus norvegicus*	NP_058747.1	65
*Homo sapiens*	NP_000627.2	64
*Mus musculus*	NP_038699.2	62
*Danio rerio*	NP_956270.1	63
*Xenopus tropicalis*	NP_001005694.1	63
*Anopheles gambiae*	XP_314490.4	61
*Drosophila melanogaster*	NP_476925.1	64
*Plutella xylostella*	PXGS_V2_005896	84
*Manduca sexta*	Msex004078-RA	89

Gene ID represents the NCBI reference sequence ID, except for *P*. *xylostella* and *M*. *sexta* which are gene IDs from their respective databases.

**Fig 1:**
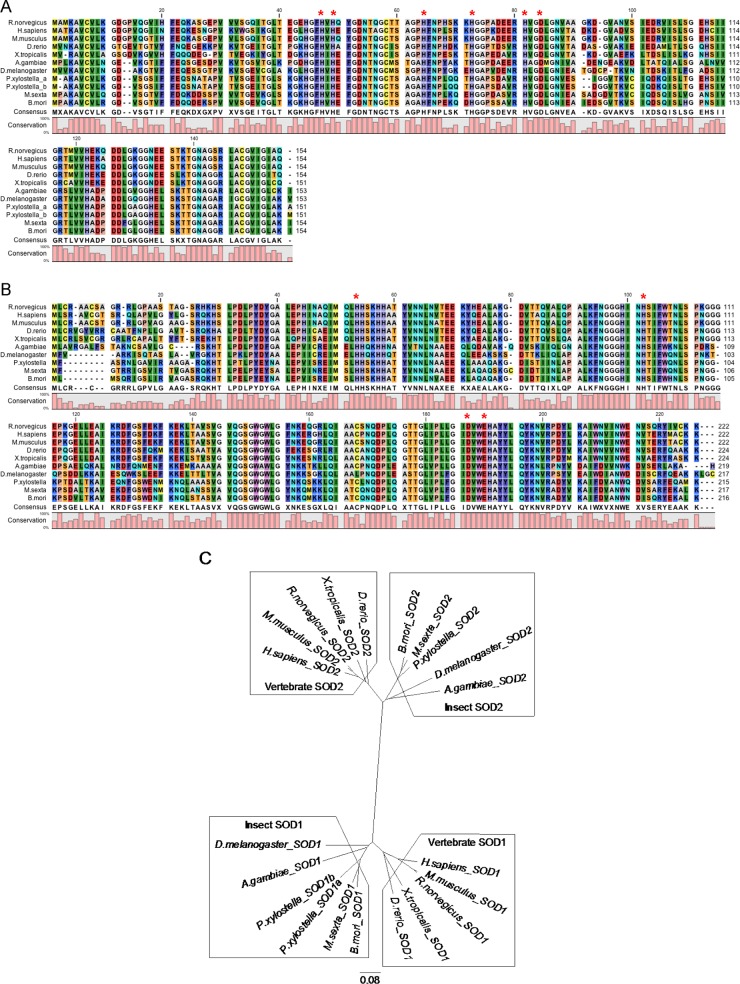
Amino acid alignment and tree of SOD1 and SOD2 from *B*. *mori* and other species. Amino acid sequence alignment of SOD1 (A) and SOD2 (B) of *B*. *mori* and other species: *Homo sapiens*, *Rattus norvegicus*, *Mus musculus*, *Danio rerio*, *Xenopus tropicalis*, *Anopheles gambiae*, *Drosophila melanogaster*, *Plutella xylostella* (a; PXGS_V2_006641, b; PXGS_V2_031456), and *Manduca sexta*. Conserved amino acids H (His), E (Glu) and D (Asp) of human SOD1 and SOD2 are marked by red asterisks (*). Levels of conserved amino acid residues among the various species are graphically shown below the sequences. Residues in the alignment are color-coded according to the Rasmol color scheme (http://life.nthu.edu.tw/~fmhsu/rasframe/COLORS.HTM#aminocolors) (C) Cluster tree for SOD1 and SOD2 of *B*. *mori* and other species.

Alignment of the deduced BmSOD1 and BmSOD2 amino acid sequences and SOD1 and SOD2 orthologs from other species showed that the BmSOD1 protein sequence contains all of the conserved His and Asp residues (Fig. 1-A, red asterisks). In addition, the BmSOD2 protein sequence contains all of the conserved His, Asp and Glu residues (Fig. 1-B, red asterisks). These amino acid residues are involved in the coordination of the metal domain.

**Fig 2:**
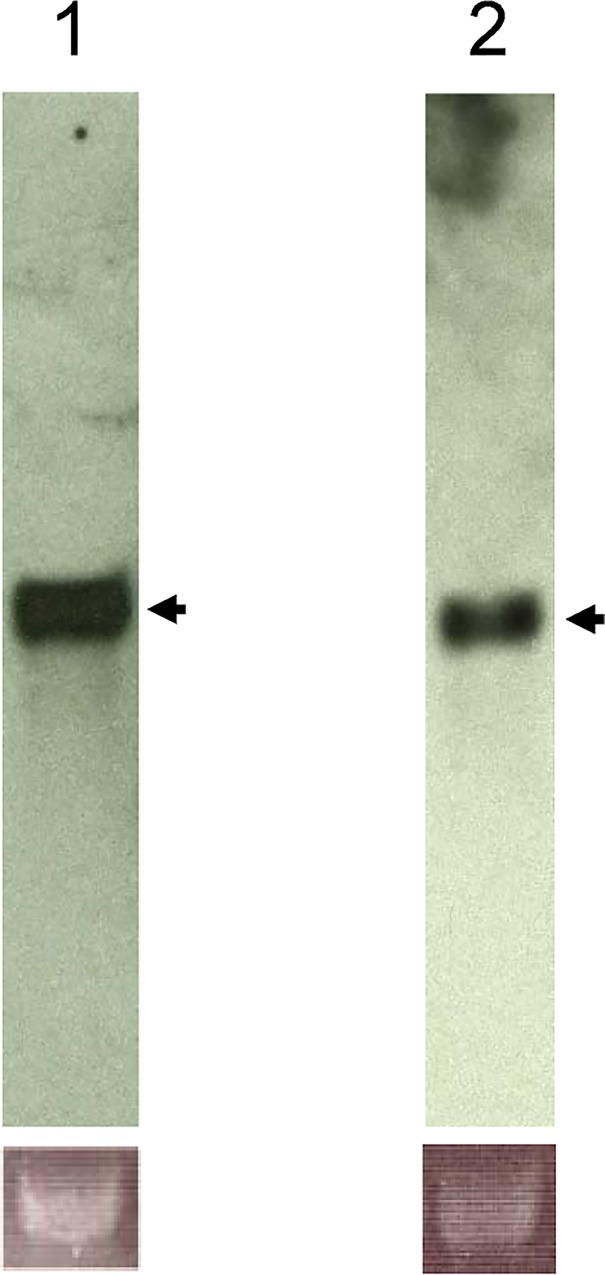
Northern blot analysis of *B*. *mori* SOD1 and SOD2. A. Total RNA (12 μg per lane) isolated from the *B*. *mori* fat body was analyzed by northern blot analysis using a probe for BmSOD1 and BmSOD2. A band at about 936 bases was identified as the BmSOD1 transcript (1), while a band at about 922 bases was identified as the BmSOD2 transcript (2). Total RNA was loaded for BmSOD1, BmSOD2 and lower panel of 18S rRNA (control of RNA loading).

In the phylogenetic tree, vertebrate SOD1 and SOD2 and insect SOD1 and SOD2 were placed into four distinct clusters (Fig. 1-C). Northern blot analysis revealed that both transcription products were single products with characteristic sizes: 936 bases for BmSOD1 and 922 bases for BmSOD2 (Fig. 2).

### Specificity of antibodies against BmSOD1 and BmSOD2

**Fig 3:**
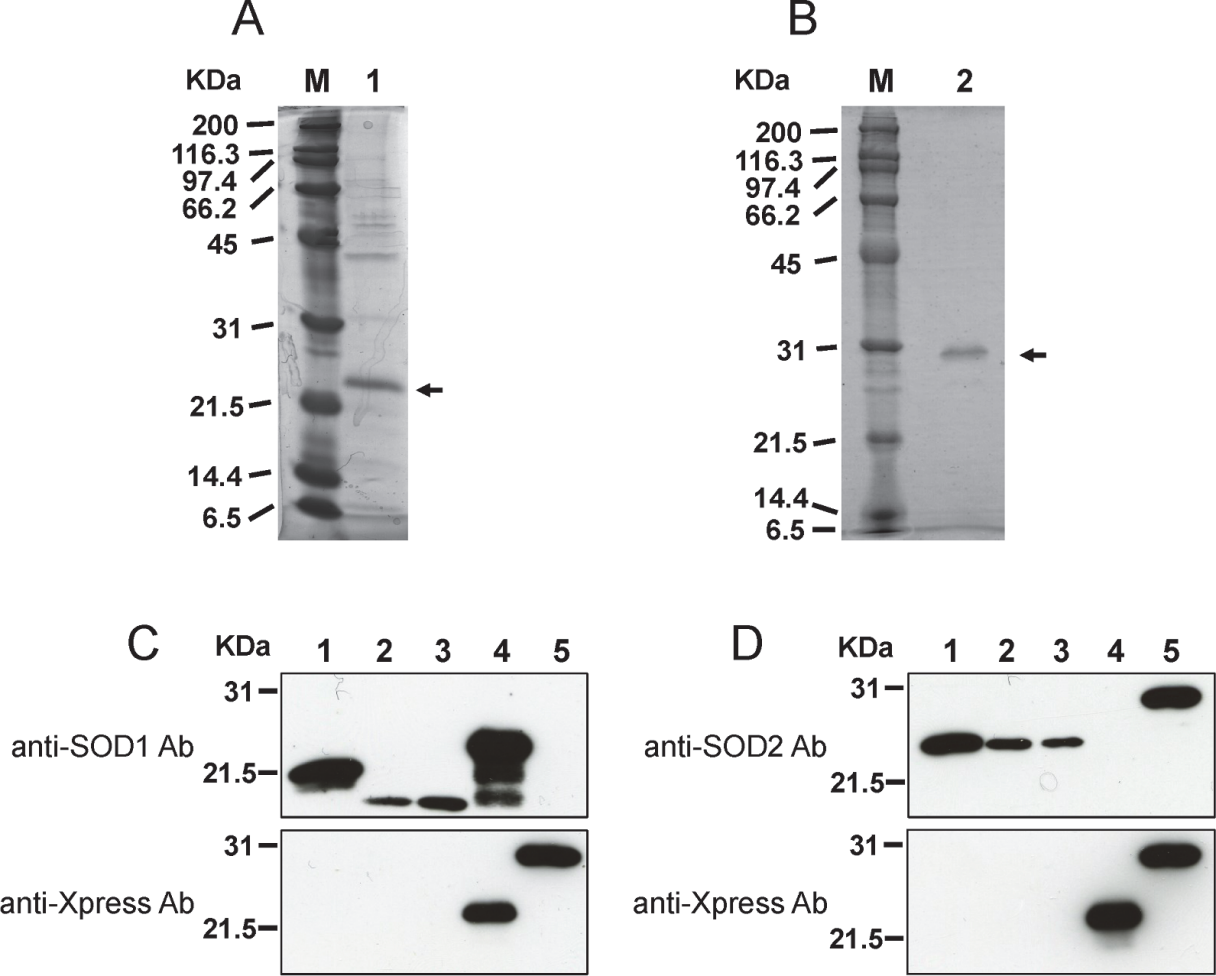
Specificity of anti-SOD antibodies. Recombinant BmSOD1 (A) and BmSOD2 (B) were separated on a 12% SDS-PAGE gel, transferred onto a nitrocellulose membrane, and processed for immunoblotting with anti-SOD antibodies and anti-Xpress antibody. Specificity of anti-SOD antibodies and anti-Xpress antibodies (C and D) was tested on the following samples (by lane): 10 μg of HeLa cell lysate as a positive control (lane 1); 10 μg of BmN4 lysate (lane 2); 10 μg of testis lysate (lane 3); 0.5 μl of recombinant BmSOD1 protein (lane 4); and0.1 μl of recombinant BmSOD2 protein (lane 5). These samples were tested against anti-SOD1 antibody (C, Upper panel), anti-SOD2 antibody (D, Upper panel) and anti-Xpress antibody (C and D, Lower panel).

Based on the conserved evolution of amino acid sequences of SOD proteins, we examined the utility of commercially available antibodies raised against human SOD1 and rat SOD2 to identify BmSOD1 and BmSOD2. Anti-SOD-1 antibody reacted with recombinant BmSOD1 protein as a 22-kDa band and with BmSOD1 in cell and tissue lysates from *B*. *mori* as a 16-kDa band (Fig. 3-A, C). Anti-SOD-2 antibody reacted with recombinant BmSOD2 protein as a 30-kDa band and with BmSOD2 in cell and tissue lysates from *B*. *mori* as a 24-kDa band (Fig. 3-B, D). Further, SOD1 antibodies did not recognize recombinant BmSOD2, and SOD2 antibodies did not recognize recombinant BmSOD1 (Fig. 3-C, D). The molecular weight of the recombinant BmSOD1 and BmSOD2 proteins (Fig. 3-C, D lanes 4 and lane 5) was slightly greater than the endogenous BmSOD1 and BmSOD2 proteins, as these proteins included the Xpress tag protein. Thus, we concluded that the commercial antibodies were useful for immunoblotting.

### Identification of developmental stage and tissue-specific expression patterns of BmSOD1 and BmSOD2 by immunoblotting

**Fig 4:**
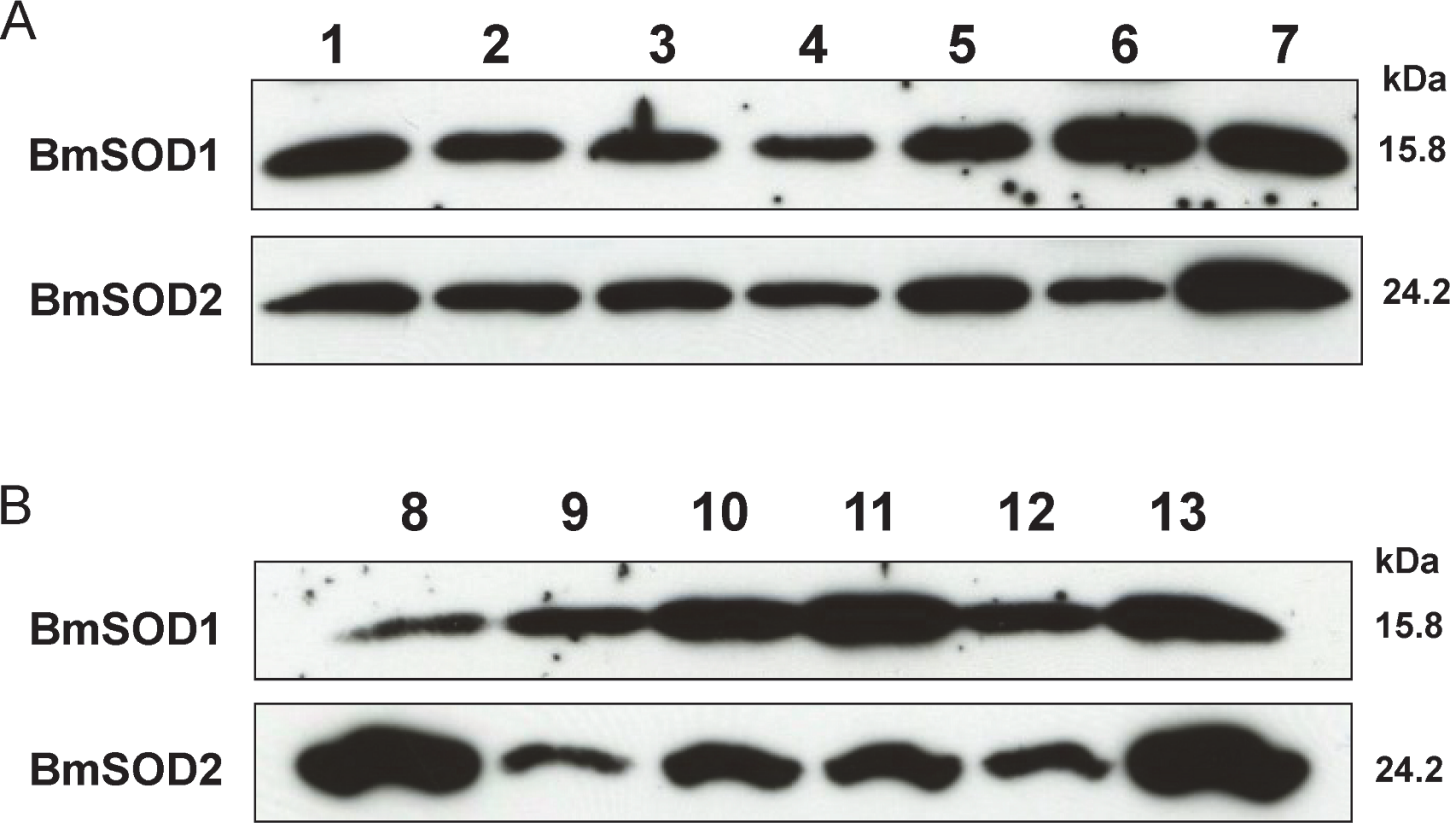
Developmental and tissue distribution of BmSODs in *B*.*mori* Aliquots (10 μg) of whole body homogenates from *B*. *mori* of the following stages, separated by SDS-PAGE, transferred to nitrocellulose and probed with anti-SOD antibodies (A): day 0 of the first (lane 1), second (lane 2), third (lane 3), fourth (lane 4) and fifth (lane 5) instar larvae, as well as pupae (lane 6) and adult (lane 7) stages. Aliquots (10 μg) of protein from various tissues of day 3 fifth instar larvae were subjected to SDS-PAGE and were examined for expression of both BmSODs antibodies (B): midgut (lane 8), silk gland (lane 9), testis (lane 10), fat body (lane 11), ovary (lane 12) and Malpighian tubule (lane 13).

**Fig 5:**
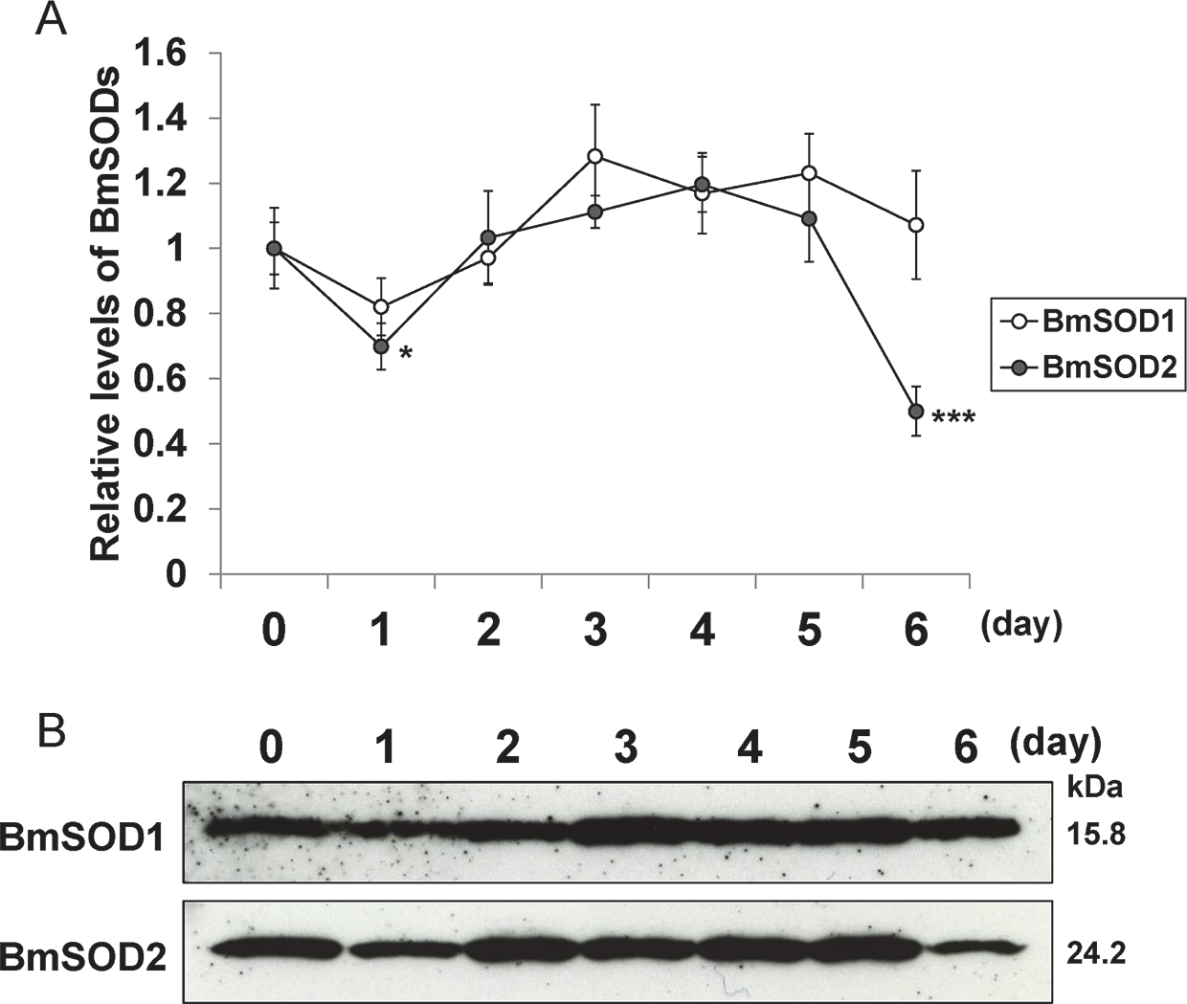
Relative BmSOD1 and BmSOD2 protein levels in the fat body of fifth instar larvae. Relative expression levels (mean±SE; n = 3) of both BmSOD proteins plotted as a line graph (A) based on aliquots (10 μg) of protein samples from the fat body in day 0 to 6 fifth instar larvae, and SDS-PAGE with anti-SOD1 and anti-SOD2 antibodies for fifth instar larvae (B) at day 0 (lane 1), day 1 (lane 2), day 2 (lane 3), day 3 (lane 4), day 4 (lane 5), day 5 (lane 6) and day 6 (lane 7). Statistically significant differences against day 0 values determined by Student’s t-test are indicated as *, P<0.05 and ***, P<0.001.

Distribution of BmSOD1 protein and BmSOD2 protein expression by developmental stage and tissue is shown in Fig. 4. Both BmSOD proteins were expressed in the whole body through all developmental stages (Fig. 4 and S2-A Fig.). In addition, BmSOD proteins were expressed in various tissues from day 3 fifth instar larvae, but BmSOD2 protein expression levels were higher in the midgut and Malpighian tubules than in other tissues (Fig. 4 and S2-B Fig.). To determine the distribution pattern of BmSODs, we studied the fat body from day 0 to 6 fifth instar larvae by immunoblotting. BmSOD proteins were expressed throughout the larval developmental stages in the fat body, but BmSOD2 protein expression was low in day 1 and 6 fifth instar larvae (Fig. 5-A, B and S3 Fig.).

### BmN4 cells treated with ROT, ISDN and detection of BmSODs

Next, we investigated the effect of chemical factors on the expression of BmSODs. We used ROT and ISDN as ROS generators, as these chemicals induced oxidative stress on BmN4 cells.

**Fig 6:**
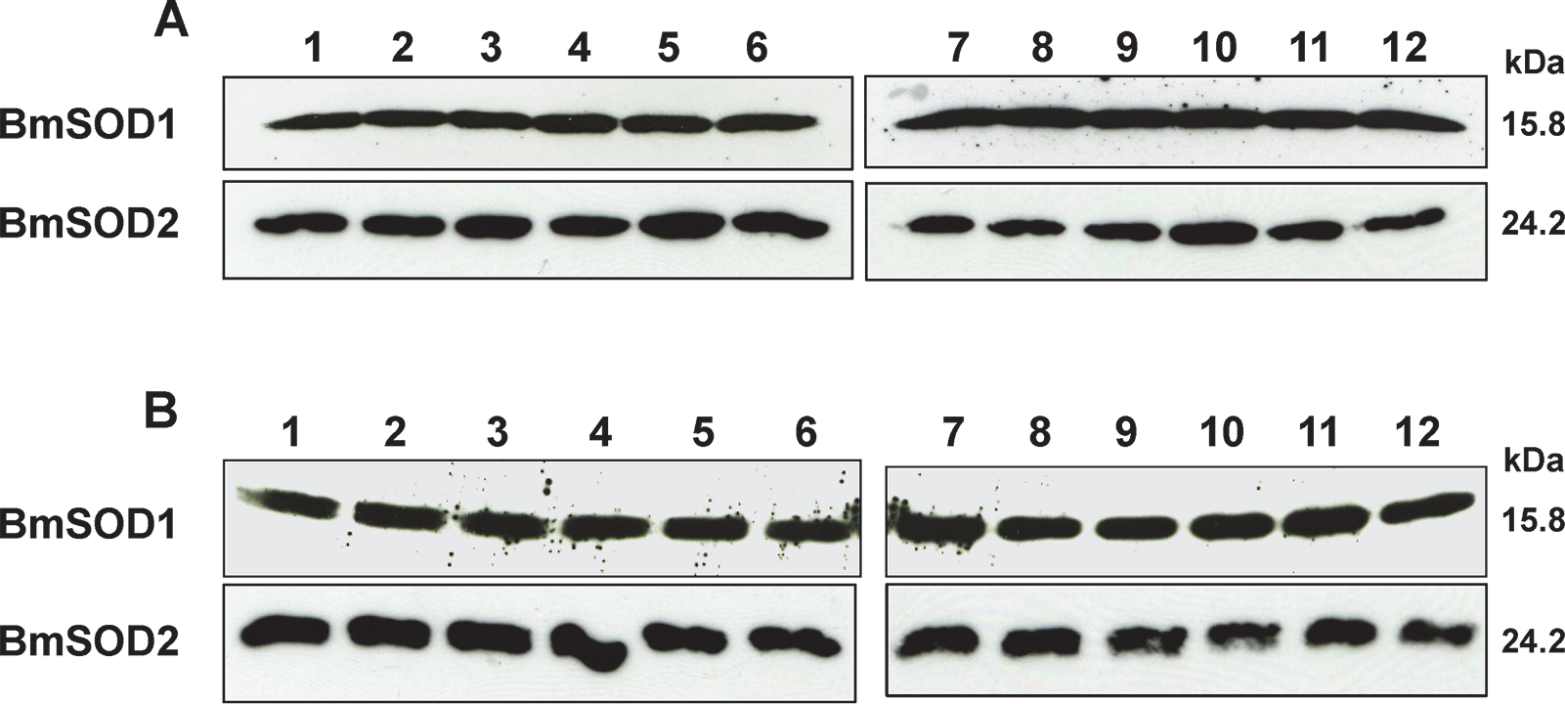
Effects of ROT- and ISDN-induced oxidative stress on BmSOD1 and BmSOD2 expression in BmN4 cells. BmN4 cells exposed to ROT for 3 or 6 hours (A) or ISDN for 3 or 6 hours (B) were examined for BmSOD1 and BmSOD2 content by immunoblotting. Aliquots (10 μg) of protein samples from BmN4 cells subjected to the following treatments: control (lanes 1–3) and ROT treatment (lane 4–6) for 3 hours; control (lane 7–9) and ROT treatment (lane 10–12). All samples were separated by SDS-PAGE, transferred to nitrocellulose and probed with each specific antibody.

Expression levels of both BmSOD proteins were the same for exposure to ROT for 3 and 6 hours (Fig. 6-A and S4 Fig.). With exposure to ISDN, a nitric oxide (NO) generator [23], expression levels of ISDN-treated BmN4 cells were the same as for the control (Fig. 6-B and S5 Fig.). Thus, ROT and ISDN did not affect the expression levels of either BmSOD protein (Fig. 6 A, B).

### BmSOD response to UV irradiation at mRNA and protein level in *B*. *mori* larvae

**Fig 7:**
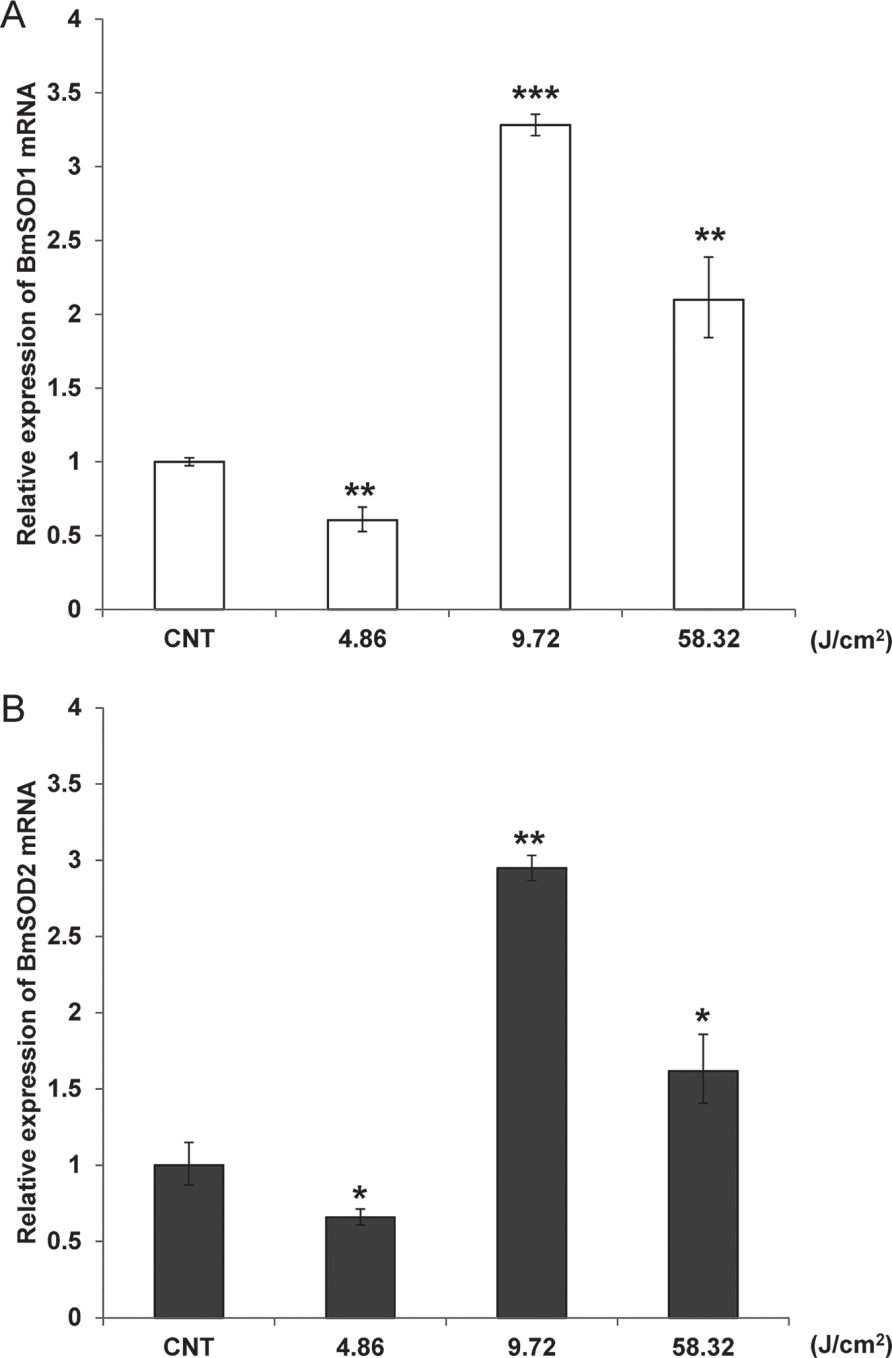
Quantitative RT-PCR confirmation of BmSOD1 and BmSOD2 expression in UV-irradiated fat body. (A) BmSOD1, (B) BmSOD2. mRNA expression in fat bodies pooled from larvae subjected to UV-irradiation at 4.86 J/cm^2^ (n = 3), 9.72 J/cm^2^ (n = 3), 58.32 J/cm^2^ (n = 4) and non-irradiated control (n = 5) was plotted as RQ values. Error bars indicate the relative minimum/maximum expression levels against mean RQ expression levels. Technical replication was performed triplicate. Statistical significance was determined by Student’s t-test. *, P<0.05; **, P<0.01; and ***, P<0.001 compared with control values.

**Fig 8:**
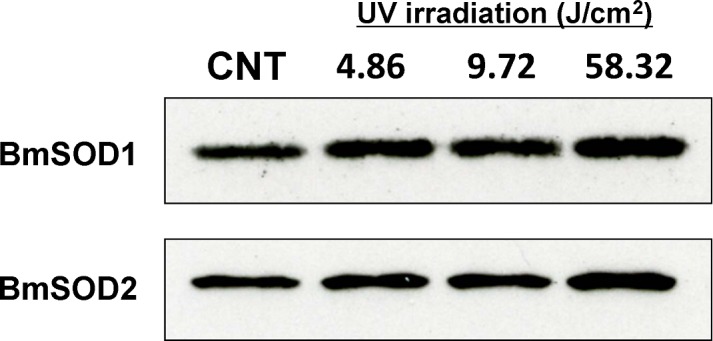
Expression levels of BmSOD1 and BmSOD2 proteins in fat body treated with UV irradiation. Fat bodies from UV-irradiated and non-irradiated (control) larvae were assessed. Aliquots (10 μg) of protein samples from fat body of day 3 fifth instar larvae were separated by SDS-PAGE, transferred to nitrocellulose, and probed with anti-SOD1 and anti-SOD2 antibodies: non-irradiated (lane 1; CNT), and irradiated at 4.86 J/cm^2^ (lane 2), 9.72 J/cm^2^ (lane 3), and 58.32 J/cm^2^ (lane 4).

Examination of expression levels of mRNA for both BmSOD by qRT-PCR after UV irradiation at 4.86, 9.72 and 58.32 J/cm^2^ showed that BmSOD1 and BmSOD2 mRNA expression was significantly increased in the 9.72 J/cm^2^ group (Fig. 7), while protein expression levels of BmSOD1 and BmSOD2 were slightly increased in the UV irradiation groups (Fig. 8 and S6 Fig.).

To examine the differences in mRNA level due to UV irradiation, we performed microarray analysis. Gene set enrichment analysis by DAVID of genes in larvae without and with UV irradiation showed distinct features in gene functions. Genes up-regulated after 6 and 12 hours of UV irradiation were present at significant level in the insulin signaling (Table 4-A) and PPAR signaling pathways (Table 4-C), respectively.

**Table 4:** Gene Set Enrichment Analysis of up/down-regulated genes after UV irradiation.

(A) 6 hours/up-regulated genes			
KEGG ID	KEGG PATHWAY	Count	P-Value
hsa04910	Insulin signaling pathway	10	0.017668
hsa00051	Fructose and mannose metabolism	6	0.018265
hsa00052	Galactose metabolism	5	0.038165
hsa04370	VEGF signaling pathway	5	0.04827
hsa00010	Glycolysis / Gluconeogenesis	7	0.060706
hsa05110	Vibrio cholerae infection	6	0.062223
(B) 6 hours/down-regulated genes			
KEGG ID	KEGG PATHWAY	Count	P-Value
hsa05414	Dilated cardiomyopathy	9	9.65E-06
hsa05410	Hypertrophic cardiomyopathy (HCM)	9	1.01E-04
hsa04260	Cardiac muscle contraction	8	0.003906
hsa04670	Leukocyte transendothelial migration	7	0.016766
hsa00980	Metabolism of xenobiotics by cytochrome P450	5	0.027911
hsa00982	Drug metabolism	5	0.027911
hsa00480	:Glutathione metabolism	6	0.041439
hsa05412	Arrhythmogenic right ventricular cardiomyopathy (ARVC)	4	0.06068
hsa04510	Focal adhesion	9	0.068888
hsa05416	Viral myocarditis	4	0.077825
(C) 12 hours/up-regulated genes			
KEGG ID	KEGG PATHWAY	Count	P-Value
hsa00561	Glycerolipid metabolism	7	0.020019
hsa03320	PPAR signaling pathway	7	0.040499
hsa04020	Calcium signaling pathway	7	0.059621
hsa00330	Arginine and proline metabolism	7	0.083331
(D) 12 hours/down-regulated genes			
KEGG ID	KEGG PATHWAY	Count	P-Value
hsa05110	Vibrio cholerae infection	7	0.018686
hsa04621	NOD-like receptor signaling pathway	4	0.048372
hsa00970	Aminoacyl-tRNA biosynthesis	7	0.071722
hsa04612	Antigen processing and presentation	4	0.082363

KEGG PATHWAY IDs and corresponding descriptions by P-values are shown for up/down-regulated genes at 6 and 12 hours after UV irradiation. Count refers to the number of genes associated with the KEGG PATHWAY IDs. P-values are based on EASE Score, and modified Fisher Exact P-values were calculated using the DAVID web server (http://david.abcc.ncifcrf.gov/).

Moreover, genes up-regulated and down-regulated after 6 hours of UV irradiation contained statistically significant Gene Ontology (GO) terms and included those involved in protein localization (GO:0008104) and oxidative stress response (GO:0006979), respectively (S1-A and B Table).

## Discussion

In this study, we characterized the expression and distribution of two BmSOD proteins in *B*. *mori*. Both SODs had highly conserved amino acid sequences. Characterization of the *B*. *mori* homologs BmSOD1 and BmSOD2 by cDNA cloning from the fat body of fifth instar larvae confirmed the presence of His and Asp, which are key functional residues in both SODs (Fig. 1-A, B). On a phylogenetic tree of SOD proteins, vertebrate SODs and insect SODs placed in distinct clusters (Fig. 1-C). These results show that insect SODs may have distinct functions from vertebrate SODs. Each BmSOD was well conserved across the species and also, each BmSOD might have a distinct function. In mammals, SOD1-deficient mice appear normal at birth but show increasing levels of oxidative stress over time [24], while SOD2-knockout mice can only live for a short span (10 days) before showing cardiomyopathy [25], as SOD2 responds to numerous inflammatory and oxidative stimuli [26].

In insects, SOD1-null flies show markedly decreased lifespans and are highly susceptible to oxidation [27]. Diapause-associated protein 3 has a copper/zinc superoxide dismutase (Cu/Zn-SOD) domain and is involved in the regulation of diapause in *Antheraea pernyi* [28]. *Hyphantria cunea* SOD1 and SOD2 play a role in resistance to oxidative stress, and SOD2, in particular, plays a role with apo lipophorin III in apoptosis [29,30]. *Apis cerana* SOD2 transcript levels fluctuate across developmental stages and are involved in development, growth and environmental stress responses [31]. Thus, insect SODs have physiological functions that confer resistance to some types of oxidative stress that are common across various species but that also play species-specific roles.

Both BmSODs exist as single isoforms that map to different chromosomes in *B*. *mori*. Accordingly, *B*. *mori* SOD1 and SOD2 might have different roles within cells. Previous studies indicated that BmSOD1 and BmSOD2 mRNA are expressed in various tissues in day 3 fifth instar larvae [16,17] with BmSOD2 mRNA being more strongly expressed in the testis. In our current study, both SOD proteins were expressed in the whole body throughout all developmental stages, and BmSOD1 protein was expressed uniformly in various tissues in day 3 fifth instar larvae. BmSOD2 protein expression levels were higher in the midgut and Malpighian tubules compared to levels in other tissues.

The fat body is an important organ with endocrine and storage functions similar to the vertebrate liver [32]. Thus, we examined developmental distribution of both BmSOD proteins in the fat body of fifth instar larva. BmSOD2 protein expression level was dramatically decreased in the fat body of day 6 fifth instar larvae.

To identify the factors that regulate expression levels of BmSOD1 and BmSOD2 proteins, we used two chemical agents that generate ROS. First, BmN4 cells were treated with ROT, which inhibits the mitochondrial electron transfer chain of mitochondrial complex I. ROT enhances the production of ROS and reduces the production of ATP, resulting in mitochondrial dysfunction [4]. However, expression of BmSOD1 and BmSOD2 proteins did not change following treatment with ROT. Then, BmN4 cells were treated with ISDN, a NO generator, and similarly, ISDN did not affect the protein expression levels of BmSOD1 and BmSOD2 (Fig. 6).

In contrast to the observation for the chemical induction of oxidative stresses, UV-irradiated BmN4 cells showed marked mRNA level changes for both BmSODs (data not shown) as was previously observed for mammalian SODs [33]. UV exposure was found to generate oxidative stress, to produce DNA damage and to rapidly enhance the activity of SOD in *Helicoverpa armigera* adults [34]. Consequently, examination of the physiological function of BmSOD1 and BmSOD2 proteins in *B*. *mori* larvae following UV irradiation using qRT-PCR and immunoblotting showed that the mRNA expression level of both BmSODs was significantly elevated in the 9.72 and 58.32 J/cm^2^ UV irradiation groups, but protein expression levels were only slightly increased in *B*. *mori* larvae treated with 58.32 J/cm^2^ UV irradiation. Expression levels of BmSOD1 and BmSOD2 mRNA were not matched to protein expression.

Microarray analysis showed that genes up-regulated after 6 hours of UV irradiation predominantly contained insulin signaling pathway related genes (Table 4-A). Moreover, genes down-regulated after 6 hours of UV irradiation predominantly contained GO terms for oxidative stress response- related genes (S1-B Table). In this study, BmSOD1 and BmSOD2 mRNA expression levels were markedly up-regulated but protein expression levels were almost unaltered in the UV irradiated groups. These observations suggest that the translational efficiency of BmSOD1 and BmSOD2 may be decreased, or that the speed of destructive metabolism in BmSOD1 and BmSOD2 protein may be increased by protein phosphorylation. In fact, we found that many genes coding for protein kinase and phosphatase were included in the insulin signaling pathway that was significantly up-regulated after 6 hours of UV irradiation based on microarray analysis (data not shown). Therefore, expressed BmSOD1 and BmSOD2 proteins may be rapidly degraded in the body of *B*. *mori*.

*B*. *mori* larvae are known to accumulate uric acid (UA) as quite a urate granules, which causes a whitening of integument color [35], and UA plays a powerful role as a physiological antioxidant and in protecting individuals from environmental stress factors [36]. In the case of UV irradiation to the whole body of *B*. *mori* larvae, UA in the integument may also have played a role in providing protection against UV irradiation stress. Both BmSODs may play protective roles against UV irradiation stress. Accordingly, we will examine the relation between UA and BmSODs in the future studies.

There is another candidate biological factor that controls the expression level of BmSOD1 and BmSOD2 in *B*. *mori*-growth factors. Ecdysteroid and juvenile hormone (JH) titers are up-regulated in day 6 of fifth instar larvae hemolymph in *M*. *sexta* and *B*. *mori* [37,38]. Furthermore, JH esterase (JHE) plays an important role in the control of hemolymph JH titer and the induction of metamorphosis, and JHE mRNA expression is detected in the fat body from day 4 to 7 fifth instar larvae [39]. Also, insulin receptor substrate (IRS) and single insulin receptor (InR), which operate in the insulin/insulin growth factor signaling (IIS) pathway, are up-regulated in the fatbody of day 6 fifth instar *B*. *mori* larvae [39]. In addition, InR and IRS mRNA levels were found to be elevated by treatment with 20-hydroxyecdysone in *B*. *mori* [40]. Thus, it is possible that BmSOD2 is down-regulated in the fat body on the last day of the larvae stage by growth factors such as hormones. This suggests that BmSOD2 plays some role in pupation. However, we were unable to clarify whether BmSOD1 and BmSOD2 were related to these hormones due to the application of oxidative stress through the application of ISDN or ROT or UV irradiation in this study. Consequently, the distinct expression patterns in the tissues for these two SODs during the final larval stage suggest that each SOD may play distinct roles in the larval developmental stage.

In summary, we characterized two *B*. *mori* SOD proteins and their different roles in the fat body in response to environmental stress and in the metamorphosis from larval to pupal stages in *B*. *mori*. These results suggest that BmSOD1 and BmSOD2 modulate environmental oxidative stress in the cell and might serve as metamorphosis-related proteins in *B*. *mori*. In future studies, we plan to further investigate the advanced molecular mechanisms in pupation *B*. *mori* in vivo using a gene knockout approach.

## Supporting Information

S1 Fig *B*. *mori* larvae were treated with ultraviolet (UV) irradiation. (A): Cardboard box (30 cm L × 23 cm W × 30 cm D) wrapped in aluminum foil, and with two stands at the side to hold two UVL-56 lamps in place at the top of the box. Silkworm larvae were held in a 10 placed on the inside of the box for UV exposure. (B): Aluminum foil was placed over the lamps at the top of the box during UV irradiation.
(TIF)

S2 Fig SDS-PAGE and CBB staining of fifth instar larvae. Whole body homogenates from the following stages were separated by SDS-PAGE, transferred to nitrocellulose and stained with Coomassie brilliant blue staining (CBB) (A): day 0 larvae of first (lane 1), second (lane 2), third (lane 3), fourth (lane 4) and fifth (lane 5) instars, as well as pupae (lane 6) and adults (lane 7). Protein samples isolated from day 3 fifth instar larvae were analyzed the same way as in (A) in the following tissues: midgut (lane 8), silk gland (lane 9), testis (lane 10), fat body (lane 11), ovary (lane 12) and Malpighian tubule (lane 13).
(TIF)

S3 Fig SDS-PAGE and CBB staining of fifth instar larvae. Protein samples from the fat body on the following days in fifth instar larvae were separated by SDS-PAGE, transferred to nitrocellulose and stained with CBB: day 0 (lane 1), day 1 (lane 2), day 2 (lane 3), day 3 (lane 4), day 4 (lane 5), day 5 (lane 6) and day 6 (lane 7). BmSOD1 and BmSOD2 protein expression levels for these samples are shown in Fig. 5.
(TIF)

S4 Fig SDS-PAGE and CBB staining of ROT-treated BmN4 cells. Protein samples from BmN4 cells in the following ROT exposure experiments were separated by SDS-PAGE, transferred to nitrocellulose and stained with CBB: experiment 1–3, control for 3 hour (lane 1–3), experiment 1–3, ROT treatment for 3 hour (lane 4–6); experiment 1–3, control for 6 hour (lane 7–9), experiment 1–3, ROT treatment for 6 hour (lanes 10–12). Expression levels of BmSOD1 and BmSOD2 proteins for these samples are shown in Fig. 6A.
(TIF)

S5 Fig SDS-PAGE and CBB staining of ISDN-treated BmN4 cells. Protein samples from BmN4 cells in the following ISDN exposure experiments were separated by SDS-PAGE, transferred to nitrocellulose and stained with CBB: experiment 1–3, control for 3 hour (lanes 1–3), experiment 1–3, ISDN treatment for 3 hour (lanes 4–6); Experiment 1–3, control for 6 hour (lanes 7–9), experiment 1–3, ISDN treatment for 6 hour (lanes 10–12). Expression levels of BmSOD1 and BmSOD2 proteins for these samples are shown in Fig. 6B.
(TIF)

S6 Fig SDS-PAGE and CBB staining of non-irradiated and UV-irradiated larvae. Protein samples from the fat body in day 3 fifth instar larvae from the following UV irradiation treatments were separated by SDS-PAGE, transferred to nitrocellulose and stained with CBB: non-irradiated (lane 1; CNT), and UV-irradiated at 4.86 J/cm^2^ (lane 2), 9.72 J/cm^2^ (lane 3) or 58.32 J/cm^2^ (lane 4). Expression levels of BmSOD1 and BmSOD2 proteins for these samples are shown in Fig. 8.
(TIF)

S1 Table (DOC)

## References

[1] Suzuki T, Takashima T, Izawa N, Watanabe M, Takeda M (2008) UV radiation elevates arylalkylamine N-acetyltransferase activity and melatonin content in the two-spotted spider mite, *Tetranychus urticae* . J Insect Physiol 54:1168–1174 10.1016/j.jinsphys.2008.06.005 18634790

[2] Rauen U, Polzar B, Stephan H, Mannherz HG, de Groot H (1999) Cold-induced apoptosis in cultured hepatocytes and liver endothelial cells: mediation by reactive oxygen species. FASEB J 13:155–168. 9872940 10.1096/fasebj.13.1.155

[3] Bove J, Prou D, Perier C, Przedborski S (2005) Toxin-induced models of Parkinson’s disease. NeuroRx 2: 484–494. 16389312 10.1602/neurorx.2.3.484 PMC1144492

[4] Morin B, Davies MJ, Dean RT (1998) The protein oxidation product 3,4-dihydroxyphenylalanine (DOPA) mediates oxidative DNA damage. Biochem J 330:1059–1067. 9494069 10.1042/bj3301059 PMC1219245

[5] Dean RT, Fu S, Stocker R, Davies MJ (1997) Biochemistry and pathology of radical-mediated protein oxidation. Biochem J 324:1–18. 9164834 10.1042/bj3240001 PMC1218394

[6] Parkes TL, Hilliker AJ, Phillips JP (1999) Motorneurons, reactive oxygen, and life span in *Drosophila* . Neurobiol Aging 20:531–535. 10638526 10.1016/s0197-4580(99)00086-x

[7] Fridovich I (1975) Superoxide dismutases. Annual Review of Biochemistry 44: 147–159 1094908 10.1146/annurev.bi.44.070175.001051

[8] Miller AF (2012) Superoxide dismutases: ancient enzymes and new insights. FEBS Lett 586:585–595. 10.1016/j.febslet.2011.10.048 22079668 PMC5443681

[9] Mita K, Kasahara M, Sasaki S, Nagayasu Y, Yamada T, et al (2004) The genome sequence of silkworm, *Bombyx mori* . DNA Res 11: 27–35. 15141943 10.1093/dnares/11.1.27

[10] Tomita M, Munetsuna H, Sato T, Adachi T, Hino R, et al (2003) Transgenic silkworms produce recombinant human type III procollagen in cocoons. Nat Biotechnol 21: 52–56. 12483223 10.1038/nbt771

[11] Wang F, Ma S, Xu H, Duan J, Wang Y, et al (2013) High‑efficiency system for construction and evaluation of customized TALENs for silkworm genome editing. Mol Genet Genomics 288:683–690. 24077893 10.1007/s00438-013-0782-4

[12] Xia Q, Zhou Z, Lu C, Cheng D, Dai F, et al (2004) A draft sequence for the genome of the domesticated silkworm (*Bombyx mori*). Science 306: 1937–1940. 15591204 10.1126/science.1102210

[13] Xia Q, Cheng D, Duan J, Wang G, Cheng T, et al (2007) Microarray-based gene expression profiles in multiple tissues of the domesticated silkworm, *Bombyx mori* . Genome Biol 8: R162 17683582 10.1186/gb-2007-8-8-r162 PMC2374993

[14] Suetsugu Y, Futahashi R, Kanamori H, Kadono-Okuda K, Sasanuma S, et al (2013) Large scale full-length cDNA sequencing reveals a unique genomic landscape in a lepidopteran model insect, *Bombyx mori* . G3: Genes, Genomes, Genetics 3 9:1481–1492. 10.1534/g3.113.006239 23821615 PMC3755909

[15] Isobe M, Kai H, Kurahashi T, Suwan S, Pitchayawasin-Thapphasaraphong S, et al (2006) The molecular mechanism of the termination of insect diapause, part 1: A timer protein, TIME-EA4, in the diapause eggs of the silkworm *Bombyx mori* is a metallo-glycoprotein. Chembiochem 10:1590–1598. 16952188 10.1002/cbic.200600138

[16] Yamamoto K, Zhang P, Banno Y, Fujii H, Miake F, et al (2005) Superoxide dismutase from the silkworm, *Bombyx mori*: sequence, distribution, and overexpression. Biosci Biotechnol Biochem 69:507–14. 15784978 10.1271/bbb.69.507

[17] Yamamoto K, Zhang P, He N, Wang Y, Aso Y, et al (2005) Molecular and biochemical characterization of manganese-containing superoxide dismutase from the silkworm, *Bombyx mori* . Comp Biochem Physiol B Biochem Mol Biol 142:403–409. 16236537 10.1016/j.cbpb.2005.09.002

[18] Tabunoki H, Ono H, Ode H, Ishikawa K, Kawana N, et al (2013) Identification of key uric acid synthesis pathway in a unique mutant silkworm *Bombyx mori* model of Parkinson's disease. PLOS One 8:e69130 10.1371/journal.pone.0069130 23894418 PMC3722175

[19] Huang DW, Sherman BT, Lempicki RA (2009) Systematic and integrative analysis of large gene lists using DAVID bioinformatics resources. Nature Protoc 4:44–57. 10.1038/nprot.2008.211 19131956

[20] Huang DW, Sherman BT, Lempicki RA (2009) Bioinformatics enrichment tools: paths toward the comprehensive functional analysis of large gene lists. Nucleic Acids Res 37: 1–13. 10.1093/nar/gkn923 19033363 PMC2615629

[21] Towbin H, Staehelin J, Gordon J (1979) Electrophoretic transfer of proteins from polyacrylamide gels to nitrocellulose sheets: procedure and some applications. Proc Natl Acad Sci USA 76: 4350–4354. 388439 10.1073/pnas.76.9.4350 PMC411572

[22] Yamamoto K, Nohata J, Kadono-Okuda K, Narukawa J, Sasanuma M, et al (2008) A BAC-based integrated linkage map of the silkworm *Bombyx mori* . Genome Biol 9:R21 10.1186/gb-2008-9-1-r21 18226216 PMC2395255

[23] Tabunoki H, Ode H, Banno Y, Katsuma S, Shimada T, et al (2011) BmDJ-1 is a key regulator of oxidative modification in the development of the silkworm, *Bombyx mori* . PLOS One 6:e17683 10.1371/journal.pone.0017683 21455296 PMC3063780

[24] Sims-Robinson C, Hur J, Hayes JM, Dauch JR, Keller PJ, et al (2013) The role of oxidative stress in nervous system aging. PLOS One 8:e68011 10.1371/journal.pone.0068011 23844146 PMC3699525

[25] Huang TT, Carlson EJ, Raineri I, Gillespie AM, Kozy H, et al (1999) The use of transgenic and mutant mice to study oxygen free radical metabolism. Ann N Y Acad Sci 893:95–112. 10672232 10.1111/j.1749-6632.1999.tb07820.x

[26] Pani G, Koch OR, Galeotti T (2009) The p53-p66shc-Manganese Superoxide Dismutase (MnSOD) network: a mitochondrial intrigue to generate reactive oxygen species. Int J Biochem Cell Biol 41:1002–1005. 10.1016/j.biocel.2008.10.011 18992840

[27] Mockett RJ, Radyuk SN, Benes JJ, Orr WC, Sohal RS (2003) Phenotypic effects of familial amyotrophic lateral sclerosis mutant Sod alleles in transgenic *Drosophila* . Proc Natl Acad Sci USA. 100:301–306. 12502789 10.1073/pnas.0136976100 PMC140958

[28] Bi Z, Yang X, Yu W, Shu J, Zhang Y (2014) Diapause-associated protein3 functions as Cu/Zn superoxide dismutase in the Chinese oak silkworm (*Antheraea pernyi*). PLOS One 9:e90435 10.1371/journal.pone.0090435 24613963 PMC3948625

[29] Kim YI, Kim HJ, Kwon YM, Kang YJ, Lee IH, et al (2010) Modulation of MnSOD protein in response to different experimental stimulation in *Hyphantria cunea* . Comp Biochem Physiol B Biochem Mol Biol 157:343–350. 10.1016/j.cbpb.2010.08.003 20728562

[30] Kim YI, Kim HJ, Kwon YM, Kang YJ, Lee IH, et al (2011) RNA interference mediated knockdown of apolipophorin-III leads to knockdown of manganese superoxide dismutase in *Hyphantria cunea* . Comp Biochem Physiol A Mol Integr Physiol 159:303–312. 10.1016/j.cbpa.2011.03.022 21458580

[31] Jia H, Sun R, Shi W, Yan Y, Li H, et al (2014) Characterization of a mitochondrial manganese superoxide dismutase gene from *Apis cerana cerana* and its role in oxidative stress. J Insect Physiol 60:68–79. 10.1016/j.jinsphys.2013.11.004 24269344

[32] Colombani J, Raisin S, Pantalacci S, Radimerski T, Montagne J, et al (2003) A nutrient sensor mechanism controls *Drosophila* growth. Cell 114:739–749. 14505573 10.1016/s0092-8674(03)00713-x

[33] Vilela FM, Fonseca YM, Jabor JR, Vicentini FT, Fonseca MJ (2012) Effect of ultraviolet filters on skin superoxide dismutase activity in hairless mice after a single dose of ultraviolet radiation. Eur J Pharm Biopharm 80:387–392. 10.1016/j.ejpb.2011.10.005 22036989

[34] Meng JY, Zhang CY, Zhu F, Wang XP, Lei CL. (2009) Ultraviolet light-induced oxidative stress: effects on antioxidant response of *Helicoverpa armigera* adults. J Insect Physiol 55:588–592. 19418599 10.1016/j.jinsphys.2009.03.003

[35] Hayashi Y (1960) Xanthine dehydrogenase in the silkworm *Bombyx mori* L. Nature 186: 1053–1054. 14400300 10.1038/1861053a0

[36] Ames BN, Cathcart R, Schwiers E, Hochstein P (1981) Uric acid provides an antioxidant defense in humans against oxidant- and radical- caused aging and cancer: A hypothesis. Proc. Natl. Acad. Sci. USA 78: 6858–6862. 6947260 10.1073/pnas.78.11.6858 PMC349151

[37] Nation JL (2008) Insect physiology and Biochemistry second edition,: CRC Press New York U.S.A 123–129p.

[38] Muramatsu D, Kinjoh T, Shinoda T, Hiruma K (2008) The role of 20-hydroxyecdysone and juvenile hormone in pupal commitment of the epidermis of the silkworm, *Bombyx mori* . Mech Dev 125:411–420. 10.1016/j.mod.2008.02.001 18331786

[39] Kamimura M, Takahashi M, Kikuchi K, Reza AM, Kiuchi M (2007) Tissue-specific regulation of juvenile hormone esterase gene expression by 20-hydroxyecdysone and juvenile hormone in *Bombyx mori* . Arch Insect Biochem Physiol 65:143–51. 17570489 10.1002/arch.20186

[40] Liu Y, Zhou S, Ma L, Tian L, Wang S, et al (2010) Transcriptional regulation of the insulin signaling pathway genes by starvation and 20-hydroxyecdysone in the *Bombyx* fat body. J Insect Physiol 56:1436–1444. 10.1016/j.jinsphys.2010.02.011 20197069

