# Supplementary figures and images for: Superoxide Dismutases, SOD1 and SOD2, Play a Distinct Role in the Fat Body during Pupation in Silkworm Bombyx mori
Source: PLoS One. 2015 Feb 25;10(2):e0116007. doi: 10.1371/journal.pone.0116007 (PMC4340916; doi:10.1371/journal.pone.0116007)

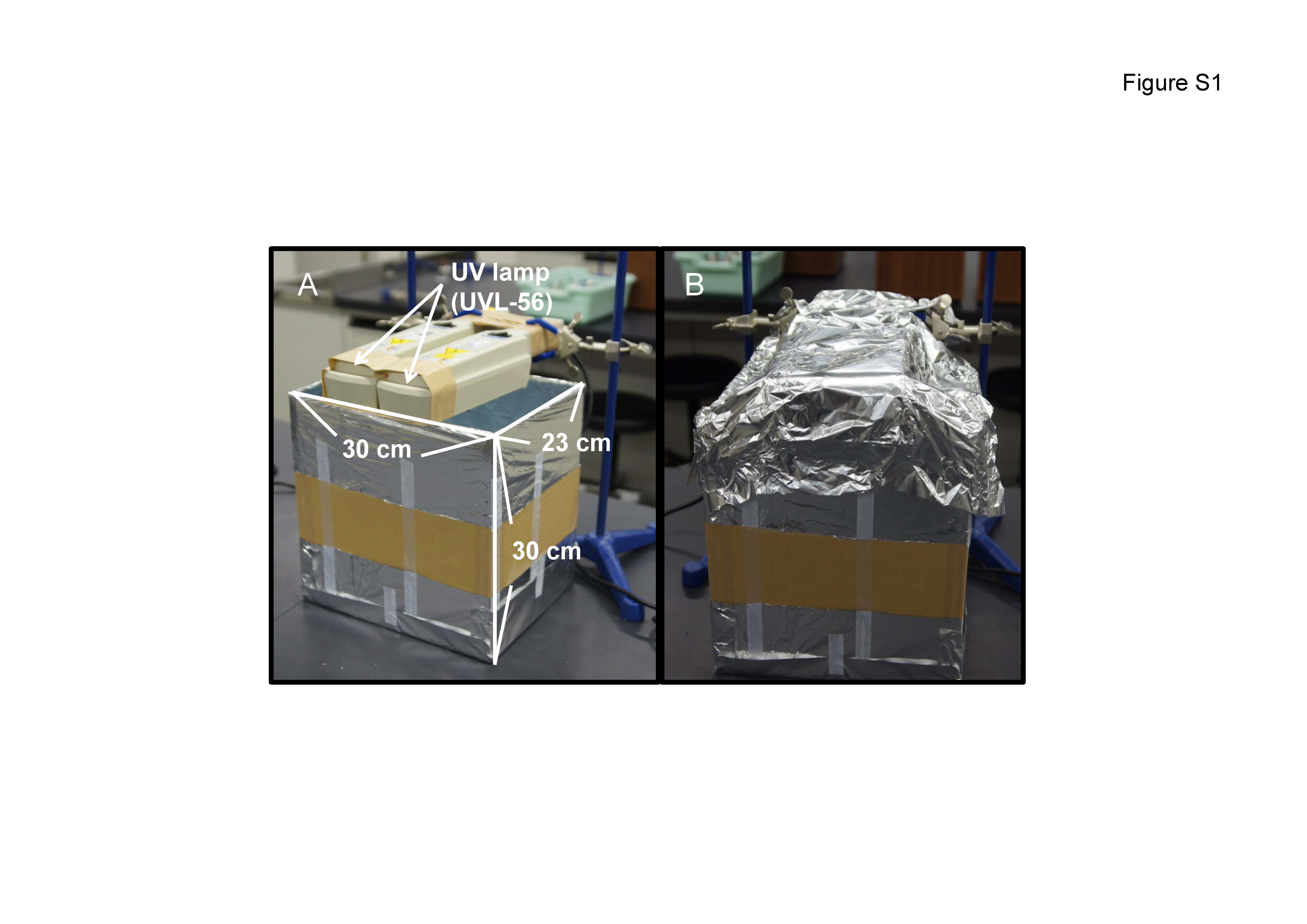

Supplement: S1 Fig — (A): Cardboard box (30 cm L × 23 cm W × 30 cm D) wrapped in aluminum foil, and with two stands at the side to hold two UVL-56 lamps in place at the top of the box. Silkworm larvae were held in a 10 placed on the inside of the box for UV exposure. (B): Aluminum foil was placed over the lamps at the top of the box during UV irradiation. (TIF) [file pone.0116007.s001.tif]

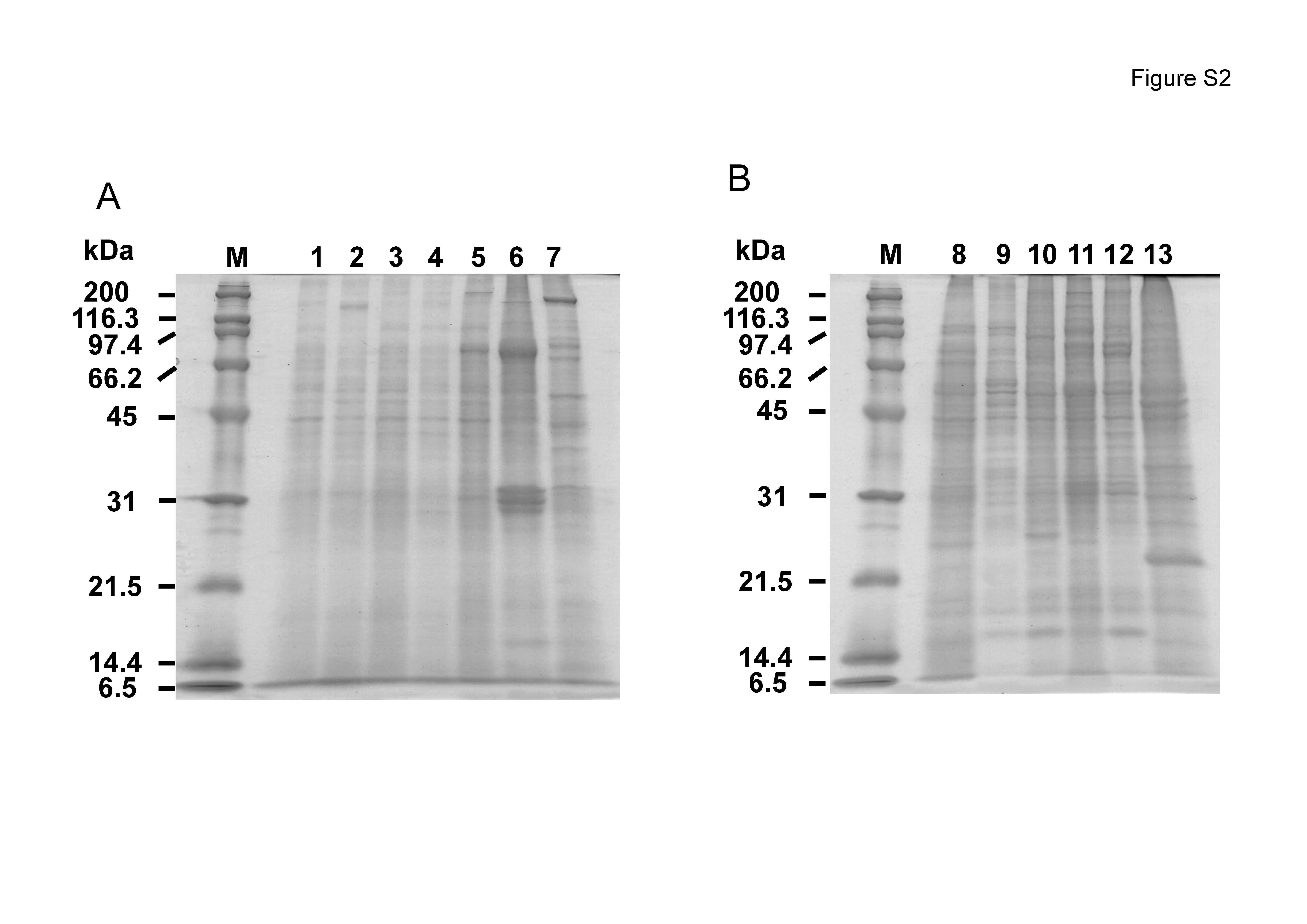

Supplement: S2 Fig — Whole body homogenates from the following stages were separated by SDS-PAGE, transferred to nitrocellulose and stained with Coomassie brilliant blue staining (CBB) (A): day 0 larvae of first (lane 1), second (lane 2), third (lane 3), fourth (lane 4) and fifth (lane 5) instars, as well as pupae (lane 6) and adults (lane 7). Protein samples isolated from day 3 fifth instar larvae were analyzed the same way as in (A) in the following tissues: midgut (lane 8), silk gland (lane 9), testis (lane 10), fat body (lane 11), ovary (lane 12) and Malpighian tubule (lane 13). (TIF) [file pone.0116007.s002.tif]

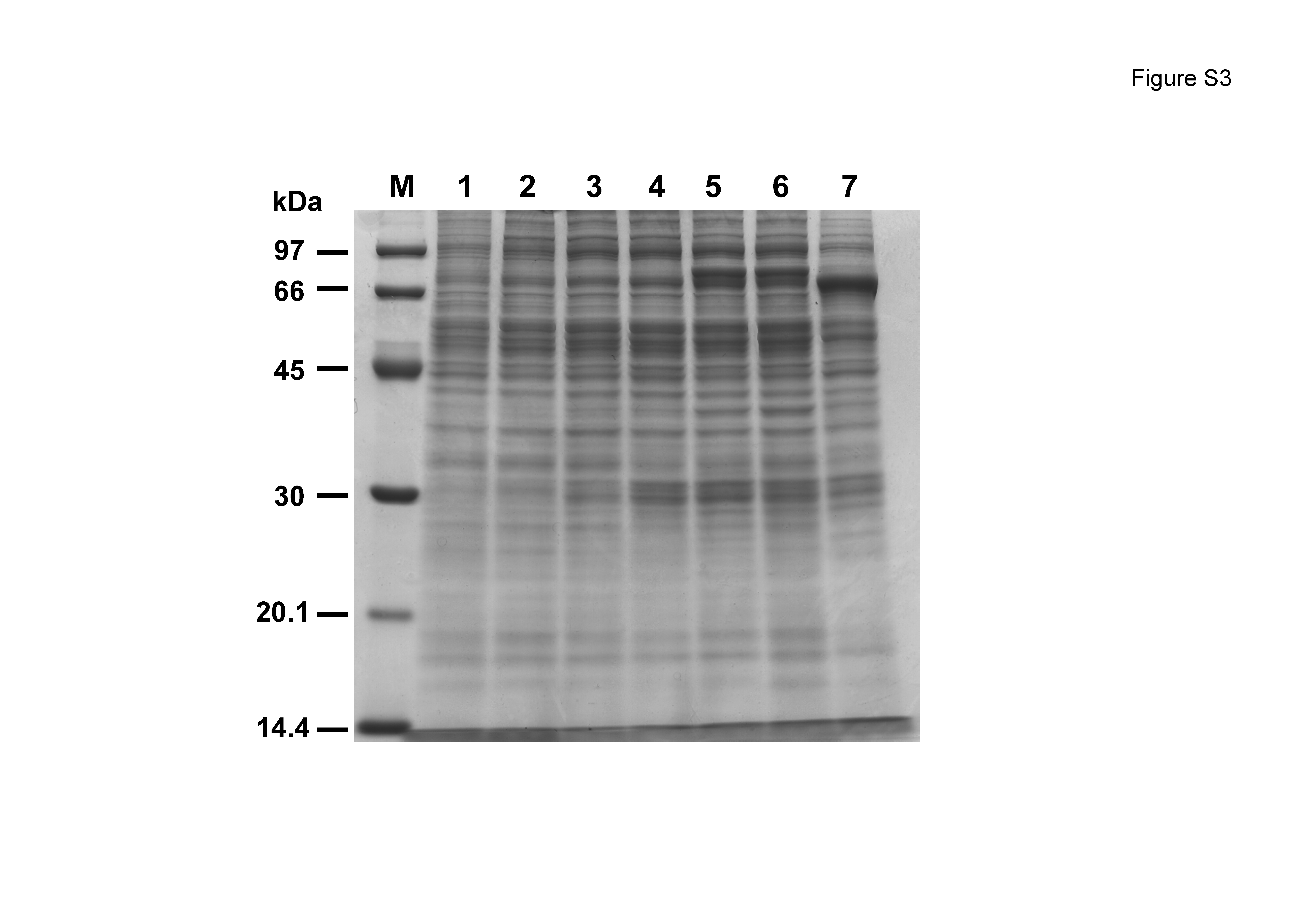

Supplement: S3 Fig — Protein samples from the fat body on the following days in fifth instar larvae were separated by SDS-PAGE, transferred to nitrocellulose and stained with CBB: day 0 (lane 1), day 1 (lane 2), day 2 (lane 3), day 3 (lane 4), day 4 (lane 5), day 5 (lane 6) and day 6 (lane 7). BmSOD1 and BmSOD2 protein expression levels for these samples are shown in Fig. 5. (TIF) [file pone.0116007.s003.tif]

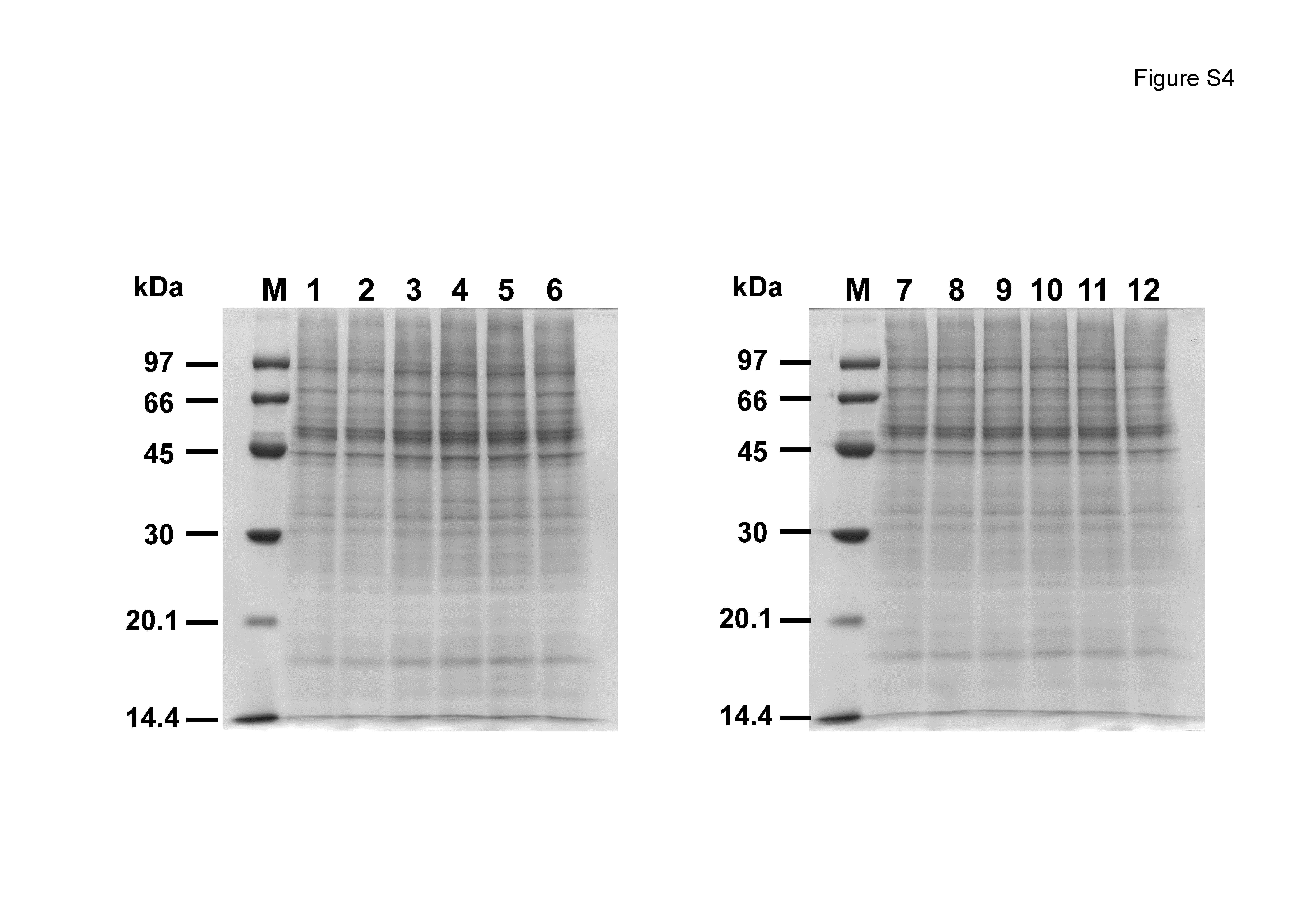

Supplement: S4 Fig — Protein samples from BmN4 cells in the following ROT exposure experiments were separated by SDS-PAGE, transferred to nitrocellulose and stained with CBB: experiment 1–3, control for 3 hour (lane 1–3), experiment 1–3, ROT treatment for 3 hour (lane 4–6); experiment 1–3, control for 6 hour (lane 7–9), experiment 1–3, ROT treatment for 6 hour (lanes 10–12). Expression levels of BmSOD1 and BmSOD2 proteins for these samples are shown in Fig. 6A. (TIF) [file pone.0116007.s004.tif]

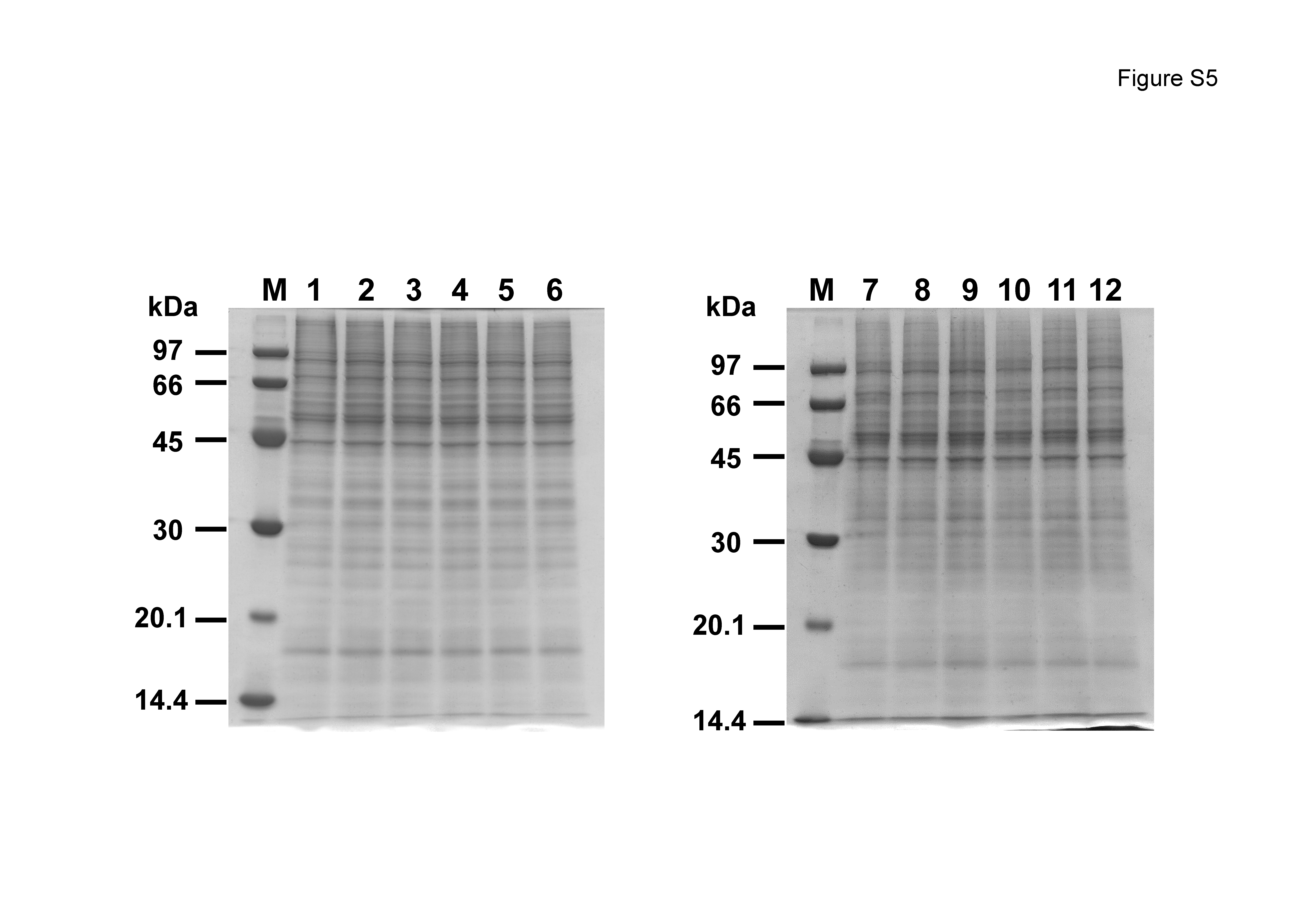

Supplement: S5 Fig — Protein samples from BmN4 cells in the following ISDN exposure experiments were separated by SDS-PAGE, transferred to nitrocellulose and stained with CBB: experiment 1–3, control for 3 hour (lanes 1–3), experiment 1–3, ISDN treatment for 3 hour (lanes 4–6); Experiment 1–3, control for 6 hour (lanes 7–9), experiment 1–3, ISDN treatment for 6 hour (lanes 10–12). Expression levels of BmSOD1 and BmSOD2 proteins for these samples are shown in Fig. 6B. (TIF) [file pone.0116007.s005.tif]

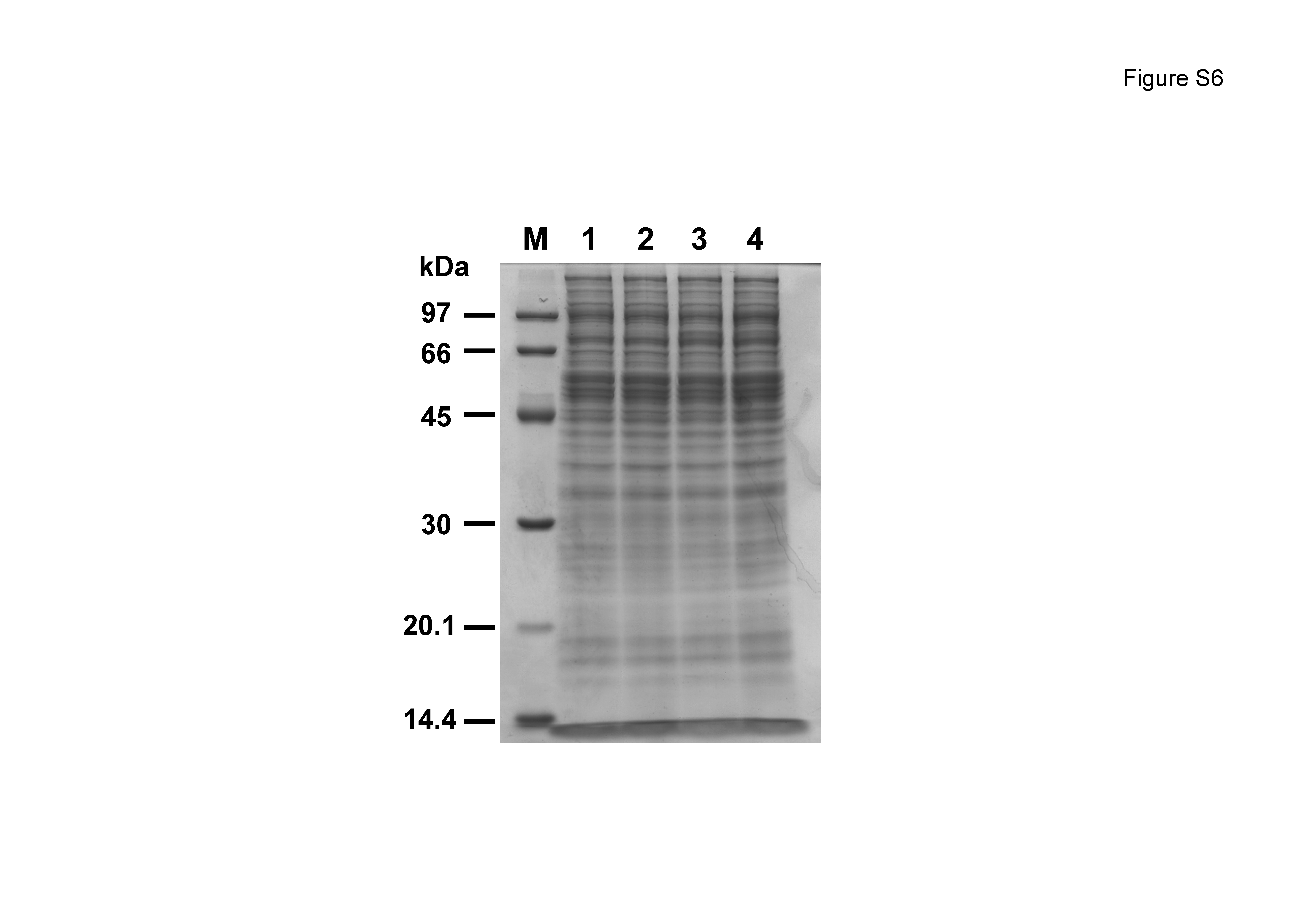

Supplement: S6 Fig — Protein samples from the fat body in day 3 fifth instar larvae from the following UV irradiation treatments were separated by SDS-PAGE, transferred to nitrocellulose and stained with CBB: non-irradiated (lane 1; CNT), and UV-irradiated at 4.86 J/cm2 (lane 2), 9.72 J/cm2 (lane 3) or 58.32 J/cm2 (lane 4). Expression levels of BmSOD1 and BmSOD2 proteins for these samples are shown in Fig. 8. (TIF) [file pone.0116007.s006.tif]
