# Supplementary material for: Superoxide Dismutases, SOD1 and SOD2, Play a Distinct Role in the Fat Body during Pupation in Silkworm Bombyx mori
Source: PLoS One. 2015 Feb 25;10(2):e0116007. doi: 10.1371/journal.pone.0116007 (PMC4340916; doi:10.1371/journal.pone.0116007)
Supplement: S1 Table — (DOC) [file pone.0116007.s007.doc]

**(A) Up-regulated genes after 6 hours**

| GO ID | GO term | Count | P |
| --- | --- | --- | --- |
| GO:0006613 | Cotranslational protein targeting to membrane | 7 | 7.3E-8 |
| GO:0006886 | Intracellular protein transport | 26 | 4.4E-7 |
| GO:0046907 | Intercellular transport | 36 | 5.2E-7 |
| GO:0006605 | Protein targeting | 19 | 7.9E-7 |
| GO:0034613 | Cellular protein localization | 26 | 2.4E-6 |
| GO:0070727 | Cellular macromolecule localization | 26 | 2.8E-6 |
| GO:0006612 | Protein targeting to membrane | 8 | 4.9E-6 |
| GO:0015031 | Protein transport | 37 | 5.7E-6 |
| GO:0045184 | Establishment of protein localization | 37 | 7.1E-6 |
| GO:0008104 | Protein localization | 39 | 2.5E-5 |

(B) Down-regulated genes after 6 hours

| GO ID | GO term | Count | P |
| --- | --- | --- | --- |
| GO:0010035 | Response to inorganic substance | 15 | 2.2E-5 |
| GO:0034614 | Cellular response to reactive oxygen species | 6 | 1.8E-4 |
| GO:0006937 | Regulation of muscle contraction | 8 | 3.2E-4 |
| GO:0000302 | Response to reactive oxygen species | 8 | 4.1E-4 |
| GO:0006979 | Response to oxidative stress | 11 | 8.1E-4 |
| GO:0044271 | Nitrogen compound biosynthetic process | 16 | 8.6E-4 |
| GO:0042743 | Hydrogen peroxide metabolic process | 5 | 9.8E-4 |
| GO:0034599 | Cellular response to oxidative stress | 6 | 1.0E-3 |
| GO:0009259 | Ribonucleotide metabolic process | 10 | 1.4E-3 |
| GO:0031032 | Actomyosin structure organization | 5 | 1.5E-3 |

(C) Up-regulated genes after 12 hours

| GO ID | GO term | Count | P |
| --- | --- | --- | --- |
| GO:0034660 | ncRNA metabolic process | 20 | 7.6E-6 |
| GO:0006414 | Translational elongation | 13 | 1.0E-5 |
| GO:0006396 | RNA processing | 33 | 1.2E-5 |
| GO:0034470 | ncRNA processing | 16 | 9.6E-5 |
| GO:0016052 | Carbohydrate catabolic process | 12 | 1.1E-4 |
| GO:0006412 | Translation | 21 | 3.6E-4 |
| GO:0006399 | tRNA metabolic process | 11 | 9.1E-4 |
| GO:0006026 | Aminoglycan catabolic process | 5 | 1.8E-3 |
| GO:0010035 | Response to inorganic substance | 14 | 2.5E-3 |
| GO:0006364 | rRNA processing | 9 | 2.5E-3 |

(D) Down-regulated genes after 12 hours

| GO ID | GO term | Count | P |
| --- | --- | --- | --- |
| GO:0006457 | Protein folding | 16 | 1.8E-6 |
| GO:0006412 | Translation | 22 | 1.9E-6 |
| GO:0006396 | RNA processing | 29 | 2.6E-6 |
| GO:0000398 | Nuclear mRNA splicing, via spliceosome | 14 | 8.6E-6 |
| GO:0000375 | RNA splicing, via transesterification reactions | 14 | 8.6E-6 |
| GO:0000377 | RNA splicing, via transesterification reactions with bulged adenosine as nucleophile | 14 | 8.6E-6 |
| GO:0016071 | mRNA metabolic process | 22 | 1.1E-5 |
| GO:0006397 | mRNA processing | 19 | 5.4E-5 |
| GO:0006399 | tRNA metabolic process | 11 | 9.7E-5 |
| GO:0006631 | Fatty acid metabolic process | 14 | 1.3E-4 |
